# Polyphenol-Enriched Black Soldier Fly Larvae (*Hermetia illucens*) Meal Improves Lipid Quality and Delays Oxidative Deterioration of Refrigerated Broiler Breast Meat

**DOI:** 10.3390/insects17090961

**Published:** 2026-09-16

**Authors:** Mustapha Kamel Fodil, Amine Ghelamallah, Said Dahmouni, Zineb Bengharbi, Abdenour Benguendouz, Djilali Benabdelmoumene, Wasim S. M. Qadi, Ahmed Mediani, Anna Kopsacheili, Konstantina Papastavropoulou, Theodoros Varzakas, Charalampos Proestos

**Affiliations:** 1 Applied Animal Physiology Lab, Abdelhamid Ibn Badis University, Mostaganem 27000, Algeriasaid.dahmouni@univ-mosta.dz (S.D.); djilali.benabdelmoumene@univ-mosta.dz (D.B.); 2 Institute of Systems Biology (INBIOSIS), Universiti Kebangsaan Malaysia, UKM, Bangi 43600, Selangor, Malaysia; 3 Laboratory of Food Chemistry, Department of Chemistry, National and Kapodistrian University of Athens Zografou, 15771 Athens, Greecedinapapa@chem.uoa.gr (K.P.); 4 Department of Food Science and Technology, University of the Peloponnese, 24100 Kalamata, Greece

**Keywords:** black soldier fly larvae, broiler, breast meat, lipid oxidation, volatile compound

## Abstract

This study investigated whether black soldier fly larvae meal (BSFL; *Hermetia illucens* L.) produced on polyphenol-rich agro-industrial by-products could enhance broiler meat lipid quality and oxidative stability during refrigerated storage. A total of 200 one-day-old Arbor Acres broilers were allocated to five dietary treatments: control diet, 5% standard full-fat BSFL meal, 10% standard full-fat BSFL meal, 10% full-fat BSFL meals produced on olive leaf and spent tea residues, and 10% defatted BSFL meal. Compared with standard full-fat BSFL meal, the olive leaf–spent tea BSFL meal showed markedly higher total phenolic content (6.74 vs. 1.82 mg GAE/g DM), DPPH radical-scavenging activity (54.80 vs. 22.40%) and ABTS antioxidant capacity (38.20 vs. 15.60 umol TE/g DM), together with lower peroxide value (1.46 vs. 2.38 meq O_2_/kg lipid) and TBARS (0.24 vs. 0.39 mg MDA/kg meal). Also, when compared with 10% standard full-fat BSFL meal, the olive leaf–spent tea BSFL meal improved final body weight (2592.8 vs. 2511.6 g) and feed conversion ratio (1.53 vs. 1.58). Breast meat from this group contained higher oleic acid (40.52 vs. 37.48%) and a-linolenic acid (1.72 vs. 1.29%), while showing a lower n-6/n-3 ratio (11.81 vs. 14.49) and thrombogenicity index (0.78 vs. 0.92). After 10 days of refrigerated storage, breast meat from birds fed the polyphenol-enriched BSFL meal showed markedly lower oxidative deterioration, as indicated by reduced TBARS values (0.60 vs. 1.12 mg MDA/kg), protein carbonyl formation (2.16 vs. 3.35 nmol/mg protein) and hexanal abundance (5.10 vs. 11.90 a.u.; internal-standard-normalized peak area). This treatment also better-preserved redness, as reflected by higher CIE values (3.52 vs. 2.40), compared to the control group. Overall, these results suggest that substrate-tailored BSFL meal may serve as a functional feed ingredient for improving the lipid quality of broilers’ breast meat and delaying oxidation-driven quality deterioration during refrigerated storage.

## Simple Summary

Black soldier fly larvae are increasingly used as an alternative ingredient in poultry feed, but their nutritional value can change depending on what the larvae are fed and how the resulting meal is processed. This study examined whether rearing the larvae on a mixture containing olive leaves and spent tea residues could improve the quality of the larvae meal and, in turn, affect chicken growth and breast meat quality. When the BSFL meal produced from larvae reared on olive leaves and spent tea residues and the standard full-fat BSFL meal were both included at 10% of the diet, the former was associated with better growth efficiency, improved meat color, lower water losses during storage and cooking, and a more favorable fat composition. It was also associated with slower deterioration of meat fats and proteins during refrigerated storage and with lower formation of compounds linked to oxidation. These findings suggest that olive leaves and spent tea residues could be useful for producing black soldier fly larvae meal with added nutritional value while also supporting the reuse of food and agricultural by-products.

## 1. Introduction

The increasing demand for sustainable animal-derived foods has intensified the search for alternative feed ingredients capable of reducing dependence on conventional protein sources while maintaining animal performance, product quality, nutritional value, and storage stability. This transition is consistent with circular bioeconomy and waste-to-feed strategies, in which organic residues and agro-industrial by-products are converted into value-added feed resources rather than treated as disposal streams [1,2,3]. In this context, insects have been increasingly considered as nutrient-dense and resource-efficient feed ingredients for sustainable animal production [4,5]. Among them, black soldier fly larvae meal (BSFL: *Hermetia illucens* L.) has attracted particular attention because of its high protein and lipid contents, valuable amino acid profile, mineral fraction, and potential bioactive compounds [1,6,7]. In poultry nutrition, experimental studies have evaluated BSFL meal as a partial substitute for soybean meal or fish meal, with generally acceptable effects on growth performance, carcass traits, and basic meat quality when dietary inclusion levels are properly formulated [8,9]. Recent reviews also place insect-derived ingredients among the most promising options for sustainable poultry feeding and product-quality-oriented nutrition [10,11]. However, the potential value of BSFL meal extends beyond conventional protein replacement, because its composition may also influence meat fatty acid deposition, oxidative stability, volatile-compound formation, and quality preservation during refrigerated storage.

A key characteristic of BSFL meal is its compositional plasticity. The protein, lipid, ash, mineral, and fatty acid profiles of larvae are influenced by the rearing substrate and larval developmental stage [12,13], whereas post-harvest processing may further affect nutrient availability and ingredient quality [14]. Therefore, BSFL meal should not be viewed as a chemically uniform feed material, but rather as a substrate-dependent biological matrix whose nutritional and functional properties can be modulated before dietary inclusion [1,2,7]. BSFL lipids are generally dominated by saturated fatty acids, particularly lauric, myristic, and palmitic acids; nevertheless, the fatty acid composition of the rearing substrate can partly modify the deposition of unsaturated lipids in larval biomass [6,12]. This is particularly relevant for poultry meat, because dietary lipid composition is a major determinant of meat fatty acid profile, nutritional lipid indices, and susceptibility to oxidative deterioration during storage [8,15].

Polyphenol-containing agro-industrial by-products, including olive leaves, olive pomace, grape pomace, tea residues, and pomegranate peel, have received attention as potential materials for insect bioconversion because their valorization may combine waste reduction with the recovery of compounds possessing antioxidant activity [16,17,18]. Nevertheless, a high polyphenol content does not in itself demonstrate that a material is an appropriate BSFL rearing substrate. Substrate composition can influence larval development, nutrient utilization, survival, biomass production, and the chemical composition of the resulting insect meal [12,13]. Accordingly, the suitability of antioxidant-rich agro-industrial residues for BSFL production should be evaluated from both larval-performance and final-meal perspectives. Olive-derived residues are of particular interest because they contain phenolic compounds such as oleuropein, hydroxytyrosol, and tyrosol, which have been associated with antioxidant and antimicrobial activities [17]. Spent tea residues may likewise retain phenolic and other bioactive compounds after beverage preparation [16,18]. Previous evidence indicates that such differences in rearing-substrate composition can contribute to variation in the nutritional and functional characteristics of BSFL biomass [19].

Plant-derived antioxidant compounds may reduce lipid and protein oxidation through radical scavenging, metal chelation, interruption of oxidative chain reactions, and protection of unsaturated lipids and heme pigments from oxidative degradation [16,18]. In poultry meat, these mechanisms are particularly relevant because oxidative reactions during refrigerated storage can promote rancidity, discoloration, off-odor development, nutritional losses, and deterioration of product quality [19,20]. Consequently, substrate-mediated changes in BSFL meal composition could potentially influence downstream meat characteristics in addition to the conventional nutritional contribution of insect meal to the diet.

Previous studies have demonstrated that dietary BSFL meal or BSFL fat can modify broiler meat composition, particularly amino acid and fatty acid profiles, without necessarily impairing major technological traits such as pH, color, cooking loss, or shear force [8,9,15]. Nevertheless, most available research has primarily focused on BSFL inclusion level, replacement of conventional feed ingredients, productive performance, carcass characteristics, and basic meat-quality indicators [4,8,9,10,11]. Thus, the use of BSFL as a poultry-feed ingredient is already relatively well investigated, whereas considerably less attention has been given to the upstream influence of the larval rearing substrate and to whether substrate-induced modifications of BSFL meal are subsequently reflected in meat quality during refrigerated storage.

This constitutes the principal research gap addressed in the present study. Although rearing substrate composition can modify BSFL chemistry [12,13], and dietary BSFL-derived ingredients can influence broiler meat composition and quality [8,9,15], few studies have directly linked these two levels within the same experimental framework. In particular, evidence remains limited regarding the relationship between antioxidant-rich rearing substrates, the resulting BSFL meal characteristics, and downstream responses involving meat fatty acid composition, nutritional lipid indices, lipid and protein oxidation, oxidation-derived volatile compounds, and color stability during refrigerated storage. Establishing this relationship is necessary to determine whether substrate-tailored BSFL meals may have functional value beyond their conventional role as alternative protein and lipid sources.

Chemical safety is also a critical consideration when BSFL are produced on heterogeneous organic residues or agro-industrial by-products. The mineral and heavy metal profiles of larvae may vary according to substrate composition, and toxic elements such as cadmium, lead, arsenic, and mercury remain important concerns for feed and food-chain applications [21,22]. Moreover, substrate composition can influence microbial dynamics and safety-related risks during the bioconversion of agro-industrial residues by *Hermetia illucens* [22]. Therefore, substrate-tailored BSFL production should be evaluated not only for nutritional and antioxidant value, but also for chemical and microbiological safety. The valorization of agro-industrial residues through BSFL production is scientifically meaningful only when the resulting insect meal combines desirable nutritional and functional characteristics with acceptable safety.

Therefore, the present study was designed to evaluate whether substrate-tailored BSFL meal could influence broiler performance and improve the lipid profile, oxidative stability, volatile compound formation, and refrigerated storage stability of broiler meat. The central hypothesis was that the incorporation of polyphenol-rich agro-industrial by-products into the BSFL rearing substrate would modify the chemical and antioxidant characteristics of BSFL meal and consequently improve meat lipid quality while limiting oxidative deterioration during storage. These effects were expected to be reflected by a more favorable fatty acid profile, lower formation of oxidation-derived compounds, reduced volatile markers of rancidity, and better preservation of color stability. By integrating rearing-substrate characteristics, insect meal composition, meat lipid quality, oxidative responses, volatile compound formation, and safety assessment, this study aimed to clarify the potential functional value of substrate-tailored BSFL meal beyond its conventional role as an alternative feed ingredient.

## 2. Materials and Methods

### 2.1. Preparation and Characterization of Black Soldier Fly Larvae Meals

Black soldier fly larvae (BSFL; *Hermetia illucens* L.) meals were produced at the Applied Animal Physiology Laboratory, Faculty of Natural and Life Sciences, Abdelhamid Ibn Badis University of Mostaganem, Algeria. Three experimental meals were prepared: a standard full-fat BSFL meal, a polyphenol-rich full-fat BSFL meal, and a defatted BSFL meal. The adult BSFL colony was maintained in custom-made ventilated rearing cages (60 × 60 × 60 cm), constructed locally for experimental use (60 × 60 × 60 cm) at 28 ± 2 °C, 65 ± 5% relative humidity, and a 12 h light/12 h dark photoperiod [23]. Egg clutches were collected daily and transferred to non-commercial hatching boxes prepared for laboratory use maintained at 28 ± 1 °C and 65 ± 5% relative humidity. After hatching, neonate larvae were reared for five days on wheat bran adjusted to approximately 70% moisture before transfer to the experimental substrates.

Two substrates were prepared on a dry matter basis. The standard substrate consisted of 70% wheat bran and 30% mixed vegetable residues composed of carrot peels, potato peels, and leafy vegetable residues in equal proportions. The polyphenol-rich substrate consisted of 50% wheat bran, 25% dried olive leaf powder, and 25% dried spent green tea (*Camellia sinensis*) residues, consistent with the green-tea by-product previously used by our research group [24]. Olive leaves were collected during autumn, a period associated with high phenolic content and antioxidant potential [25]. Olive leaves and spent green tea residues were incorporated as polyphenol-rich agro-industrial materials because rearing-substrate composition can influence the nutritional and functional characteristics of BSFL biomass [26]. All plant residues were cleaned, dried at 45 °C to constant weight, ground, passed through a 1 mm sieve, homogenized, and adjusted to approximately 70% moisture before feeding.

Larval fattening was conducted in plastic trays measuring 60 × 40 × 15 cm. Each tray received 10,000 five-day-old larvae, corresponding to approximately 4.2 larvae/cm^2^, and 12 independent trays were prepared for each substrate. The feeding rate was 100 mg fresh substrate/larva/day, equivalent to approximately 1 kg/tray/day and 14 kg/tray over the 14-day fattening period [27]. Substrate was supplied every 48 h to limit compaction, overheating, and anaerobic fermentation and maintained at a depth of approximately 3–5 cm. Larvae were reared at 27 ± 2 °C and 65 ± 5% relative humidity and harvested after 14 days at the late larval stage before visible prepupal darkening.

At harvest, larvae were separated from residual substrate and frass using stainless-steel sieves, washed, drained, and weighed. Fresh biomass yield was determined from the harvested larval mass, whereas residual substrate and frass were weighed to estimate substrate reduction. Representative larvae from each tray were used to determine dry matter content, and dry matter yield was calculated from the fresh biomass yield and the corresponding dry matter proportion. Larval survival, fresh biomass yield, dry matter yield, and substrate reduction were recorded at tray level as production-performance indicators.

Immediately after harvest, larvae were blanched in hot water at 95 °C for 3 min, drained, and dried in a ventilated oven (Memmert GmbH, Schwabach, Germany) at 65 °C for 24 h to limit enzymatic and microbial deterioration [28,29]. Dried larvae were cooled to room temperature, ground, passed through a 1 mm sieve, and homogenized. Standard full-fat and polyphenol-rich full-fat BSFL meals were obtained from larvae reared on the standard and olive leaf–green tea substrates, respectively. Meals from replicate trays within each substrate were pooled after processing to obtain one homogeneous batch per meal type for experimental diet manufacturing. Defatted BSFL meal was produced from a portion of the standard full-fat meal by Soxhlet extraction with n-hexane for 6 h, followed by solvent removal, drying at 40 °C for 12 h, grinding, and sieving.

Representative samples from each homogenized meal batch were stored at −20 °C until analysis. Dry matter was determined by oven drying at 105 °C to constant weight, ash by incineration at 550 °C, and crude fat gravimetrically after Soxhlet extraction. Crude protein was determined by Kjeldahl digestion followed by automatic distillation–titration using a Büchi system (Büchi Labortechnik AG, Flawil, Switzerland), according to procedures previously applied by our research group [24,30]. Crude protein was calculated using a nitrogen-to-protein conversion factor of 6.25, whereas corrected protein was additionally estimated using the factor 4.76, as proposed for *H. illucens* and other edible insects [31]. Chitin was estimated by sequential acid demineralization and alkaline deproteinization [32].

Total lipids were extracted using chloroform:methanol (Sigma-Aldrich Chemie GmbH, Taufkirchen, Germany) (2:1, *v*/*v*) according to Folch et al. [33] and procedures previously applied by our research group [24]. Fatty acids were converted to fatty acid methyl esters (FAME) and analyzed by gas chromatography–mass spectrometry (GC–MS) using an Agilent 7890B gas chromatograph (Agilent Technologies, Santa Clara, CA, USA) equipped with a DB-23 capillary column. Fatty acids were identified by comparison with authentic FAME standards and expressed as percentages of total identified fatty acids [24,33].

Primary lipid oxidation was assessed by peroxide value using an iodometric procedure [34] and expressed as meq O_2_/kg lipid. Secondary lipid oxidation was determined using the TBARS assay [24,35]. Samples were centrifuged using an Eppendorf 5804 R refrigerated centrifuge (Eppendorf, Hamburg, Germany) and reacted with thiobarbituric acid in a Memmert WNB14 water bath (Memmert GmbH, Schwabach, Germany), and absorbance was measured at 532 nm using a Shimadzu UV-1800 UV–Vis spectrophotometer (Shimadzu Corporation, Kyoto, Japan). Results were expressed as mg malondialdehyde equivalents/kg meal.

Methanolic extracts were used for antioxidant analyses. The total phenolic content was determined using the Folin–Ciocalteu method [36,37], DPPH radical-scavenging activity was determined according to Brand-Williams et al. [38] and Bouhalla et al. [37], and ABTS antioxidant capacity was determined according to Re et al. [39] and Dahmouni et al. [30]. Absorbance was measured using a Jenway 6715 UV–Vis spectrophotometer (Cole-Parmer, Staffordshire, UK) at 765, 517, and 734 nm, respectively. Results were expressed as mg gallic acid equivalents (GAE)/g dry matter for total phenolics, percentage inhibition for DPPH, and µmol Trolox equivalents (TE)/g dry matter for ABTS.

Mineral and heavy-metal concentrations were determined after HNO_3_/HCl acid (Sigma-Aldrich Chemie GmbH, Taufkirchen, Germany) digestion according to the procedure described for BSFL biomass [40] and analyzed using a PerkinElmer Optima 5300 DV inductively coupled plasma optical emission spectrometer (ICP-OES; PerkinElmer, Waltham, MA, USA). Major minerals were expressed as g/kg dry matter, whereas trace elements and heavy metals were expressed as mg/kg dry matter.

### 2.2. Experimental Diets and Feeding Program

Five experimental diets differing in BSFL meal type and inclusion level were formulated as shown in Table 1. BSFL meal partially replaced soybean meal on a crude-protein-equivalent basis, while maize, vegetable oil, mineral sources, and crystalline amino acids were adjusted to maintain comparable nutrient profiles among treatments. The feeding program comprised starter (days 1–10), grower (days 11–24), and finisher (days 25–42) phases, with target metabolizable energy/crude protein values of approximately 3000 kcal/kg/22.0%, 3100 kcal/kg/20.5%, and 3200 kcal/kg/19.0%, respectively.

Standard full-fat BSFL meal was included at 5 and 10%, whereas the polyphenol-rich and defatted meals were evaluated at 10%. Comparisons among meal types therefore focused primarily on the common 10% inclusion level (T3–T5), while T2 versus T3 reflected increasing inclusion of standard full-fat BSFL rather than a formal dose–response assessment. Diets were prepared separately for each treatment and feeding phase in mash form using the same mixing procedure. No antibiotic growth promoters were added. Representative samples of each diet were analyzed for dry matter, crude protein, crude fat, ash, fatty acid profile, and mineral composition. Feed and water were provided *ad libitum* throughout the 42-day trial.

### 2.3. Birds, Housing and Experimental Design

All animal procedures were conducted in accordance with institutional guidelines for the care and use of animals in experimental research. The protocol was approved by the local ethics committee of Abdelhamid Ibn Badis University of Mostaganem, Algeria, under approval number 2024/07/41. A total of 200 one-day-old male Arbor Acres broiler chicks (initial body weight: 42.1 ± 1.8 g) were randomly assigned to five dietary treatments in a completely randomized design. Each treatment consisted of four replicate pens with ten birds per pen, giving 40 birds per treatment. The pen was considered the experimental unit for growth-performance measurements. Birds were reared for 42 days in 1.20 × 1.00 m floor pens equipped with a feeder and drinker and bedded with clean wood shavings. Temperature was maintained at 32 ± 1 °C during the first week and gradually reduced to 24 ± 2 °C during the finisher period, while relative humidity was maintained at 55–70%. The lighting program consisted of 23 h light: 1 h darkness during the first three days and 20 h light: 4 h darkness thereafter. Feed and water were provided *ad libitum*. Birds were vaccinated according to the local preventive program, and no therapeutic antibiotics were administered during the trial.

### 2.4. Growth Performance, Slaughtering and Breast Meat Sampling

Body weight was recorded at placement and at the end of each feeding phase (days 1, 10, 24, and 42). The feed intake was calculated at the pen level as feed offered minus feed residues. Body weight gain, average daily gain, average daily feed intake, and feed conversion ratio were calculated for each feeding phase and for the overall day 1–42 period. The pen was considered the experimental unit for all growth-performance variables. Mortality was recorded daily, and feed conversion ratio was corrected for mortality when necessary. At 42 days of age, two birds per replicate pen with body weights closest to the mean body weight of their respective pen were selected for slaughter, giving eight birds per treatment and 40 birds in total. The selected birds were considered subsamples nested within their respective pens and were not treated as independent experimental units. Selected birds were subjected to feed withdrawal for 10–12 h, with free access to water. Birds were individually weighed before slaughter, electrically stunned, exsanguinated, scalded, defeathered, eviscerated, washed, and drained under standardized hygienic conditions. After slaughter, carcasses were chilled at 4 °C for 24 h. The pectoralis major and thigh muscles were then excised, trimmed of visible fat and connective tissue, and identified according to treatment, replicate pen, and bird number. The pectoralis major muscle was used for meat-quality, color, oxidative-stability, and volatile-compound analyses, whereas thigh meat was used for complementary proximate-composition and fatty-acid analyses.

### 2.5. Refrigerated Storage and Meat Quality Assessment

After 24 h post-mortem chilling at 4 °C, breast and thigh muscles were excised, trimmed of visible fat and connective tissue, and labeled according to treatment, replicate pen, bird number, muscle type, and storage time. Day 0 represented the initial post-mortem assessment performed after the 24 h chilling period and was distinguished from the subsequent refrigerated-storage period. Each breast muscle was divided into separate standardized portions allocated to the different storage times; thus, the same physical meat portion was not repeatedly measured throughout storage. Portions originating from the same bird retained their common biological origin and were accounted for accordingly in the statistical analysis. For refrigerated storage, standardized breast meat portions were placed on food-grade polystyrene trays, wrapped with oxygen-permeable polyethylene film, and assigned to days 0, 3, 7, and 10, with portions for subsequent sampling times maintained at 4 ± 1 °C until analysis. Samples were randomly positioned in the refrigerator and rotated daily to minimize positional temperature effects.

At each sampling time, samples were analyzed immediately for pH and surface color. Meat pH was measured directly in the pectoralis major using a calibrated portable pH meter (HI99163, Hanna Instruments, Woonsocket, RI, USA) equipped with a penetration electrode. Color was measured after 20 min of blooming on the freshly cut surface using a calibrated colorimeter and expressed as CIE L* (lightness), a* (redness), and b* (yellowness). Measurements were taken at three points per sample, avoiding connective tissue, blood spots, and surface defects, and the mean of the three readings was retained for analysis. Drip loss was determined on standardized breast portions stored at 4 °C for 24 h and expressed as the percentage loss relative to the initial sample weight. Cooking loss was determined after cooking standardized breast portions in a water bath until an internal temperature of 75 °C was reached, followed by cooling, blotting, and reweighing, and it was expressed as the percentage weight loss relative to the initial uncooked weight.

Proximate composition was determined on homogenized breast and thigh samples. Moisture was determined by oven-drying at 105 °C to constant weight, and ash by incineration at 550 °C. Crude protein was determined by the Kjeldahl method using a nitrogen-to-protein conversion factor of 6.25 and an automatic distillation–titration system (Büchi Labortechnik AG, Switzerland), consistent with the analytical equipment reported by the same research team. Intramuscular lipid content was determined by chloroform:methanol (2:1, *v*/*v*) extraction, with the results expressed as g/100 g fresh meat. The same-team analytical procedure specifies chloroform:methanol extraction followed by phase separation and gravimetric lipid determination.

### 2.6. Fatty Acid Profile and Lipid Nutritional Indices

Total lipids were extracted from BSFL meals, diets, and meat samples using the chloroform:methanol procedure of [33]. Briefly, homogenized samples were mixed with chloroform:methanol (2:1, *v*/*v*), homogenized, and filtered. Phase separation was induced using saline solution or distilled water, and the lower organic phase was collected, evaporated under a nitrogen stream, and used for fatty acid analysis.

Fatty acids were converted into fatty acid methyl esters (FAMEs) using 14% BF_3_–methanol at 90 °C for 60 min. FAMEs were analyzed by gas chromatography coupled with flame-ionization detection (GC-FID) using an Agilent 6890N gas chromatograph (Agilent Technologies, USA) equipped with a DB-23 capillary column (60 m × 0.25 mm i.d., 0.25 µm film thickness). The oven temperature was programmed from 50 °C, held for 1 min, to 230 °C at 4 °C/min, followed by a 10 min hold at 230 °C. The injector and detector temperatures were maintained at 250 °C, and helium was used as the carrier gas at a constant flow rate of 1.0 mL/min. Individual fatty acids were identified by comparison of their retention times with those of a Supelco 37 Component FAME Mix (CRM47885; Sigma-Aldrich, St. Louis, MO, USA) and expressed as a percentage of the total identified fatty acids. These analytical conditions have been documented in previous work from the same research team.

Fatty acids were grouped as saturated fatty acids (SFA), monounsaturated fatty acids (MUFA), and polyunsaturated fatty acids (PUFA) by summing the corresponding individual fatty acids. The PUFA/SFA and n-6/n-3 ratios were calculated from the respective fatty acid sums. The atherogenicity index (AI) and thrombogenicity index (TI) were calculated according to Ulbricht and Southgate [36] as follows:

$$AI=\frac{C12:0+4(C14:0)+C16:0}{\Sigma MUFA+\Sigma n-6PUFA+\Sigma n-3PUFA}$$

$$TI=\frac{C14:0+C16:0+C18:0}{\left[0.5(\Sigma MUFA)+0.5(\Sigma n-6PUFA)+3(\Sigma n-3PUFA)+(\Sigma n-3PUFA/\Sigma n-6PUFA)\right]}$$

The peroxidability index (PI) was calculated as follows:

PI=(Σmonoenoic×0.025)+(Σdienoic×1)+(Σtrienoic×2)+(Σtetraenoic×4)+(Σpentaenoic×6)+(Σhexaenoic×8)

### 2.7. Oxidative Stability and Volatile Compound Analysis

Lipid oxidation in breast meat was evaluated at 0, 3, 7, and 10 days of refrigerated storage using the TBARS assay [35]. Homogenized samples were reacted with thiobarbituric acid under acidic conditions, and absorbance was measured at 532 nm using a Shimadzu UV-1800 spectrophotometer (Shimadzu Corporation, Kyoto, Japan). Results were expressed as mg malondialdehyde equivalents/kg meat using a 1,1,3,3-tetraethoxypropane calibration curve. Protein oxidation was determined by the 2,4-dinitrophenylhydrazine (DNPH) method [41], and the carbonyl content was calculated using an extinction coefficient of 22,000 M^−1^ cm^−1^ and expressed as nmol carbonyls/mg protein [42].

Volatile compounds were analyzed by headspace solid-phase microextraction coupled with gas chromatography–mass spectrometry (HS-SPME-GC-MS). Homogenized breast meat (3 g) was placed in 20 mL vials containing 5 mL saturated NaCl solution and 2-methylpentanal as an internal standard. Samples were equilibrated at 40 °C for 40 min, and volatiles were extracted using a 50/30 µm DVB/CAR/PDMS fiber at 40 °C for 20 min before thermal desorption at 250 °C for 2 min.

GC-MS analysis was performed using an Agilent 8890 gas chromatograph coupled to an Agilent 5977B quadrupole mass spectrometer equipped with a DB-WAX UI column (60 m × 0.25 mm i.d., 0.25 µm). Helium was used at 1.0 mL/min. Volatile compounds were identified using the NIST spectral library, retention indices, and authentic standards when available. Hexanal, pentanal, heptanal, octanal, and nonanal were monitored as oxidation-related aldehydes, and results were expressed as internal-standard-normalized peak areas.

### 2.8. Statistical Analyses

Statistical analyses were performed using SAS version 9.4 (SAS Institute Inc., Cary, NC, USA), and figures were prepared using GraphPad Prism version 11.0.2 (GraphPad Software, Boston, MA, USA). For growth-performance variables, the pen was the experimental unit (*n* = 4 pens per treatment), and dietary treatment was analyzed as a fixed effect. For meat-quality, proximate-composition, fatty-acid, and lipid-index variables, the two birds sampled from each pen were considered subsamples nested within the pen rather than independent experimental replicates. Dietary treatment was included as a fixed effect and pen as a random effect, thereby preserving the pen as the experimental unit for treatment inference. Storage-related variables were analyzed using a repeated-measures mixed model including dietary treatment, storage time (0, 3, 7, and 10 days), and treatment × storage-time interaction as fixed effects, with pen nested within treatment as a random effect. Bird nested within pen was specified as the subject for repeated measurements over storage time, accounting for the common biological origin of meat portions collected from the same bird. Tukey-adjusted comparisons were performed when significant effects were detected. BSFL meal composition was treated descriptively because each meal type represented a single pooled batch. Laboratory triplicates were analytical replicates only and were not considered independent biological replicates; therefore, no inferential comparisons were performed among BSFL meal types. Because the dietary treatments did not constitute a balanced dose–response design, treatment was analyzed as a five-level categorical factor. No overall dose–response analysis was performed, and comparisons among standard, polyphenol-enriched, and defatted BSFL meals were interpreted primarily at the common 10% inclusion level. Model residuals were assessed for normality and homogeneity of variance. Pearson correlation analysis and principal component analysis (PCA) were used to examine relationships among fatty-acid composition, lipid indices, oxidation markers, color traits, and volatile compounds [43]. Results are presented as least-square means with pooled SEM, and statistical significance was set at *p* < 0.05.

## 3. Results and Discussion

### 3.1. Chemical Composition, Lipid Profile, Antioxidant Properties and Safety of BSFL Meals

Because each BSFL meal was produced as a pooled batch for diet manufacture, the chemical, antioxidant, oxidative, mineral, and safety data are interpreted as descriptive batch characterizations. Therefore, differences among meals are discussed as analytical and biological patterns rather than as inferential treatment effects. The chemical composition of the BSFL meals was highly dependent on the larva feeding substrate and subsequent processing procedure (Table 2). The largest difference was achieved by defatting, leading to considerable increase in crude protein content from 41.20% DM for the regular full-fat BSFL meal and 40.35% DM for the olive leaf–spent tea BSFL meal up to 58.76% DM in the defatted meal. At the same time, the amount of crude fat decreased from 34.65 and 31.82% DM in full-fat meals to 8.74% DM in the defatted meal. This suggests that defatting primarily served as a concentrating procedure, providing for the creation of protein enriched ingredient, while the full-fat meals contained a significant portion of lipids. Such variation is typical for *Hermetia illucens* larvae, whose protein and lipid content vary depending on feeding substrate composition, larval developmental stage and processing conditions [6,12,44].

The corrected protein values were lower than the crude protein values calculated using the conventional nitrogen-to-protein conversion factor of 6.25. They reached 31.37, 30.72 and 44.85% DM in the standard full-fat, olive leaf–spent tea and defatted BSFL meals, respectively. This difference confirms that the conventional factor may overestimate true protein in insect meals because part of the nitrogen is associated with chitin and other non-protein nitrogenous compounds [45]. Chitin was also highest in the defatted meal, reaching 10.18% DM compared with 7.63 and 7.95% DM in the two full-fat meals. This increase probably reflects a concentration effect after lipid extraction. From a nutritional perspective, this is important because chitin may influence protein digestibility, gut fermentation and nutrient utilization in poultry.

The olive leaf–spent tea BSFL meal showed the most distinctive functional profile. Total phenolic content increased more than threefold compared with the standard full-fat meal, from 1.82 to 6.74 mg GAE/g DM. Similarly, DPPH inhibition increased from 22.40 to 54.80%, while ABTS antioxidant capacity increased from 15.60 to 38.20 umol TE/g DM. These results indicate that the incorporation of olive leaves and spent tea residues into the larval substrate enhanced the antioxidant-related properties of the resulting BSFL meal. This effect is consistent with the richness of olive leaves and tea residues in phenolic compounds, including oleuropein, hydroxytyrosol, tyrosol and catechin-derived compounds, which are known for radical-scavenging, reducing and metal-chelating activities [16,17,18].

The improved antioxidant-related profile of the olive leaf–spent tea BSFL meal was associated with a better initial oxidative status. Peroxide value decreased from 2.38 to 1.46 meq O_2_/kg lipid compared with the standard full-fat BSFL meal, while TBARS decreased from 0.39 to 0.24 mg MDA/kg meal. This suggests that the polyphenol-rich substrate not only enriched the larvae meal in antioxidant-related compounds but also limited lipid oxidation before dietary incorporation. This point is particularly relevant because oxidized dietary lipids may contribute to oxidative deterioration in meat during refrigerated storage.

The fatty acid profile of the BSFL meals was dominated by saturated fatty acids, especially lauric acid, which represented 43.60% of total fatty acids in the standard full-fat meal and 44.10% in the defatted meal. This confirms the typical lipid signature of BSFL, which is generally characterized by high proportions of lauric, myristic and palmitic acids [6,12]. However, the olive leaf–spent tea BSFL meal showed a moderate but nutritionally relevant shift toward a more unsaturated profile. Compared with the standard full-fat meal, oleic acid increased from 14.20 to 17.10%, linoleic acid from 8.70 to 9.55%, and a-linolenic acid from 0.55 to 0.77%. Consequently, this meal showed lower total SFA and higher MUFA and PUFA proportions than the standard full-fat meal. These findings support the concept that BSFL fatty acid composition can be partially modulated through substrate tailoring.

The mineral profile also differed among BSFL meals. The defatted meal showed higher ash and phosphorus contents, whereas the olive leaf–spent tea BSFL meal contained higher magnesium, iron, copper and manganese than the standard full-fat meal. These differences likely reflect both the mineral composition of the rearing substrate and the concentration effect associated with lipid removal. From a meat-quality perspective, this result is relevant because some trace elements may contribute to antioxidant enzyme systems, whereas others, particularly iron and copper, can also act as pro-oxidants when present at excessive levels.

The safety profile of the three BSFL meals was satisfactory. Lead, cadmium, arsenic and mercury remained low and did not differ significantly among meals. Lead ranged from 0.082 to 0.095 mg/kg DM, cadmium from 0.041 to 0.052 mg/kg DM, arsenic from 0.030 to 0.036 mg/kg DM and mercury from 0.006 to 0.007 mg/kg DM. These values indicate that the use of olive leaves and spent tea residues did not increase toxic metal contamination under the present production conditions. This is an essential point because insect meals produced on agro-industrial by-products must be evaluated not only for nutritional value and functional potential, but also for chemical safety [21,22]. Overall, the olive leaf–spent tea BSFL meal differed from the standard full-fat meal by its higher phenolic content, stronger antioxidant capacity, lower initial lipid oxidation and slightly improved unsaturated fatty acid profile, without evidence of increased heavy metal contamination. Therefore, this ingredient should not be interpreted only as an alternative protein or lipid source, but rather as a substrate-tailored functional BSFL meal with potential relevance for improving the oxidative stability and nutritional quality of broiler meat.

### 3.2. Growth Performance, Mortality and Viability

The effects of dietary BSFL meal inclusion on broiler growth performance from day 1 to day 42 are presented in Table 3. Initial body weight did not differ among treatments (*p* = 0.914), confirming the homogeneity of the experimental groups at the beginning of the trial. At day 42, the dietary treatment significantly affected final body weight (*p* = 0.047), body weight gain (*p* = 0.045), average daily gain (*p* = 0.045), and feed conversion ratio (*p* = 0.038). Birds fed the 10% olive leaf–spent tea BSFL meal (T4) showed significantly higher final body weight, body weight gain, and average daily gain than birds fed the 10% standard full-fat BSFL meal (T3). The remaining treatments showed intermediate values and did not differ significantly from either T3 or T4.

The average daily feed intake was not significantly affected by dietary treatment and ranged from 92.88 to 93.84 g/day. Thus, the higher growth performance observed in T4 relative to T3 was not associated with greater voluntary feed consumption. Previous broiler studies have shown that BSFL meal can be incorporated at moderate inclusion levels without compromising feed intake or productive performance when diets are adequately balanced for energy, amino acids, and digestible nutrients [8,9,46,47].

Feed conversion ratio was significantly affected by dietary treatment, with T4 showing the lowest value (1.53 ± 0.02) and T3 the highest (1.58 ± 0.02). Because the average daily feed intake remained similar among treatments, the lower FCR observed in T4 relative to T3 was primarily associated with its greater body weight gain rather than increased feed consumption. Previous evidence indicates that broiler responses to BSFL meal depend on several factors, including inclusion level, nutrient balance, amino acid adequacy, metabolizable energy estimation, and processing form [46]. Accordingly, the present results suggest that differences in BSFL meal characteristics may contribute to variation in feed efficiency, although the underlying mechanisms were not directly investigated.

The comparison between T3 and T4 is particularly relevant because both treatments contained 10% full-fat BSFL meal, allowing the two meal types to be compared at the same inclusion level. The olive leaf–spent tea BSFL meal was characterized by higher phenolic content, stronger antioxidant capacity, lower initial lipid oxidation and a slightly more favorable unsaturated fatty acid profile than the standard full-fat BSFL meal. These compositional differences may have contributed to the better growth response observed in T4; however, digestibility, intestinal morphology, systemic antioxidant status, and other metabolic responses were not directly measured. Consequently, these associations should not be interpreted as evidence of a causal mechanism.

BSFL-based diets have previously been associated with changes in intestinal health, cecal microbiota, short-chain fatty acid production, immune-related markers, and nutrient utilization, depending on inclusion level, processing method, and bird physiological status [47,48,49,50]. Such mechanisms could potentially contribute to differences in productive responses among BSFL meals. Nevertheless, gut morphology, microbiota, and antioxidant enzyme activities were not evaluated in the present study, and the higher performance of T4 should therefore be interpreted as an overall response to the dietary treatment rather than evidence of a specific intestinal or metabolic pathway.

The defatted BSFL meal group showed intermediate growth performance. Final body weight, body weight gain, average daily gain, and FCR in T5 did not differ significantly from the control or most other BSFL treatments, indicating that 10% defatted BSFL meal did not impair broiler growth under the present dietary conditions. Similar findings have been reported in studies showing that defatted or partially defatted BSFL meal can partially replace conventional protein sources in poultry diets, provided that amino acid balance, energy density and chitin-related digestibility constraints are considered [8,9,46,50]. However, the present results do not demonstrate that defatting itself improved or reduced performance relative to the other BSFL processing approaches.

Mortality remained low and was not significantly affected by dietary treatment. Viability ranged from 95.00 to 97.50%, indicating that none of the tested BSFL-containing diets adversely affected bird survival under the conditions of the present experiment. This result confirms that BSFL meal inclusion up to 10% was compatible with normal broiler viability. It also agrees with recent evidence showing that moderate BSFL meal inclusion can maintain productive performance and health status in broilers or slow-growing chickens when diets are nutritionally balanced and ingredient quality is controlled [9,47,49]. Overall, BSFL meal inclusion at the tested levels did not adversely affect feed intake, mortality, or viability. At the common 10% inclusion level, the olive leaf–spent tea BSFL treatment resulted in higher growth performance and better feed conversion than the standard full-fat BSFL treatment. However, these findings should be interpreted cautiously because inferences about growth performance were based on only four replicate pens per treatment, which limits statistical power and the generalizability of relatively small treatment differences.

### 3.3. Physicochemical Quality and Proximate Composition of Meat

The effects of dietary BSFL meal on the physicochemical quality and proximate composition of breast and thigh meat are presented in Table 4. Breast meat pH measured after 24 h post-mortem chilling at 4 °C was not significantly affected by dietary treatment (*p* = 0.286), with values ranging from 5.81 to 5.85. These similar values indicate that dietary BSFL inclusion did not markedly alter post-mortem muscle acidification. Post-mortem pH is an important determinant of protein functionality, color development, and water retention in poultry meat [51]. Because pH remained comparable among treatments, the differences observed in color, drip loss, and cooking loss were unlikely to be primarily associated with differences in post-mortem acidification.

The absence of a treatment effect on pH is consistent with previous studies showing that moderate BSFL meal inclusion can be compatible with normal technological meat characteristics. Saidani et al. [9] reported no significant effects of BSFL meal inclusion on post-mortem pH, water-holding capacity, cooking loss or meat chemical composition in Arbor Acres broilers. Similarly, Baderuddin et al. [52] found that 6 and 12% BSFL meal did not adversely affect breast meat pH, drip loss or cooking loss. These findings support the compatibility of moderate BSFL inclusion with basic physicochemical meat quality when diets are appropriately formulated.

Color traits were more responsive than pH values. Breast meat from birds fed the olive leaf–spent tea BSFL meal showed lower lightness and higher redness than meat from the control and 10% standard full-fat BSFL groups. This suggests reduced surface paleness and improved red color intensity. Instrumental color is influenced by pigment chemistry, muscle structure, surface moisture and oxidative status; therefore, standardized color measurement is essential for interpreting poultry meat quality [51]. The lower lightness and greater redness observed in T4 may be associated with differences in the oxidative status of the meat; however, these baseline color measurements alone do not establish a direct antioxidant mechanism. Plant-derived antioxidants can help preserve meat color by limiting lipid and pigment oxidation during storage [18]. This interpretation is reinforced by the subsequent storage results, where the same treatment showed better redness stability and lower oxidation markers. Yellowness was not significantly affected by dietary treatment (*p* = 0.174). Thus, the tested BSFL meals did not produce a marked treatment-related change in the yellow component of breast meat color. This is relevant because insect-derived lipids may affect meat color depending on the larval substrate, pigment composition, and dietary inclusion level [15].

Drip loss and cooking loss, which were measured directly as separate physicochemical traits, were significantly affected by dietary treatment. Drip loss was lower in T4 (2.02 ± 0.22%) than in T3 (2.55 ± 0.31%), while cooking loss was also lower in T4 (22.15 ± 1.21%) than in T3 (24.68 ± 1.50%). Because both treatments contained 10% full-fat BSFL meal, this comparison indicates differences in these two technological traits between the standard and olive leaf–spent tea BSFL meals at the same inclusion level. Drip and cooking losses are technologically relevant because they influence the processing yield and characteristics of fresh and cooked poultry meat [53].

Because pH did not differ among treatments, the lower drip and cooking losses observed in T4 relative to T3 were unlikely to be explained primarily by differences in post-mortem acidification. Protein oxidation can modify protein structure, solubility, and hydration behavior [54,55]. The lower protein carbonyl values subsequently observed in T4 during refrigerated storage are consistent with a possible relationship between oxidative status and these physicochemical traits, although the present experiment does not establish a direct causal relationship.

Breast meat moisture, dry matter, and ash were not significantly affected by dietary treatment. Crude protein and intramuscular lipid contents, however, differed significantly among treatments. T4 had higher breast crude protein than T3 (23.48 vs. 22.60 g/100 g) and lower intramuscular lipid than T2 (1.34 vs. 1.70 g/100 g). Importantly, the superscript pattern does not support stating that breast lipid content in T4 was significantly lower than T3. Therefore, the interpretation should remain restricted to statistically supported pairwise differences.

A similar response was observed in thigh meat. Moisture, dry matter, and ash did not differ among treatments, whereas crude protein and intramuscular lipids were significantly affected. T4 had higher thigh crude protein than T3 (21.42 vs. 20.58 g/100 g), whereas the lowest intramuscular lipid content occurred in T1 and the highest in T2. T3, T4, and T5 showed intermediate lipid values and did not differ significantly from either extreme. These results indicate modest treatment-related changes in meat protein and lipid concentrations rather than a uniform shift toward a “leaner” meat composition. Although T4 also showed improved growth performance relative to T3, nutrient digestibility and tissue nutrient deposition were not measured; consequently, the observed compositional differences cannot be attributed directly to improved nutrient utilization. Fiorilla et al. [47] similarly reported that BSFL supplementation can support growth without compromising meat quality or consumer-relevant lipid characteristics. The defatted BSFL treatment showed generally intermediate physicochemical and proximate values. Because most T5 values shared superscripts with several other treatments, the present data do not demonstrate a specific beneficial or detrimental effect of defatting on meat physicochemical quality. Overall, dietary BSFL inclusion at the tested levels did not affect breast meat pH at 24 h post-mortem, yellowness, moisture, dry matter, or ash. At the common 10% inclusion level, T4 differed from T3 principally through higher redness, lower drip and cooking losses, and higher crude protein concentrations in breast and thigh meat. These associations suggest that BSFL meal characteristics may contribute to meat-quality responses, but they should not be interpreted as evidence of a specific antioxidant, digestive, or metabolic mechanism.

### 3.4. Fatty Acid Profile and Lipid Nutritional Indices of Breast Meat

The fatty acid profile and lipid nutritional indices of breast meat showed that dietary treatment significantly modified several individual fatty acids and lipid nutritional indices. This confirms that broiler breast meat lipid composition is responsive to BSFL meal inclusion level, lipid fraction and larval rearing substrate (Table 5). This response was expected because dietary lipid composition is one of the main determinants of fatty acid deposition in poultry tissues. In the present study, the comparison among standard full-fat, olive leaf–spent tea full-fat and defatted BSFL meals allowed the distinction between three effects: the inclusion of BSFL-derived lipids, the modification of BSFL meal quality through substrate tailoring and the reduction in lipid transfer through defatting.

Lauric acid was the clearest marker of BSFL-derived lipid incorporation into breast meat. Its concentration increased from 0.42 ± 0.05% in the control group to 1.35 ± 0.12% in the 5% standard BSFL group and 2.24 ± 0.18% in the 10% standard BSFL group. This dose-dependent increase confirms that the full-fat BSFL meal transferred part of its characteristic medium-chain saturated fatty acid profile to broiler breast meat. This agrees with de Souza Vilela et al. [8], who reported a marked increase in lauric acid in broiler breast meat with increasing dietary full-fat BSFL inclusion. The lower lauric acid value observed in the defatted BSFL group (0.75 ± 0.08%) further confirms that lipid extraction reduced the transfer of BSFL-specific fatty acids into meat.

Myristic and palmitic acids followed the same general pattern. Myristic acid was highest in the 10% standard full-fat BSFL group (1.04 ± 0.09%), and palmitic acid also reached its highest value in the same treatment (23.80 ± 0.82%). Consequently, the total SFA increased to 34.70 ± 1.02% in this group, compared with 31.40 ± 0.95% in the control group. This indicates that the standard full-fat BSFL meal shifted breast meat lipids toward a more saturated profile. This result is nutritionally relevant because lauric, myristic and palmitic acids are included in the calculation of atherogenicity and thrombogenicity indices and are generally considered less favorable than unsaturated fatty acids when evaluating the nutritional quality of meat lipids.

In contrast, the olive leaf–spent tea BSFL meal produced a more favorable unsaturated fatty acid pattern. Compared with the 10% standard full-fat BSFL group, this treatment increased oleic acid from 37.48 ± 1.10 to 40.52 ± 1.08%, linoleic acid from 18.70 ± 0.80 to 20.44 ± 0.75%, and a-linolenic acid from 1.29 ± 0.08 to 1.72 ± 0.11%. These changes indicate that substrate tailoring attenuated the saturated-fat enrichment usually associated with standard full-fat BSFL meal and promoted a more balanced lipid profile in breast meat. The higher oleic acid content is particularly relevant because oleic acid is generally considered a desirable MUFA in meat lipid quality evaluation.

The fatty acid groups confirmed this interpretation. Total SFA was highest in the 10% standard full-fat BSFL group, whereas the olive leaf–spent tea BSFL group maintained SFA close to the control level. MUFA was also better preserved in the olive leaf–spent tea group (43.94 ± 1.10%) than in the 10% standard full-fat BSFL group (41.30 ± 1.18%). PUFA values were less variable, but the n-3 PUFA fraction was significantly higher in the olive leaf–spent tea group (1.90 ± 0.12%) than in the 10% standard full-fat BSFL group (1.55 ± 0.09%). This improvement is important because the n-3 fraction contributes to the nutritional value of poultry meat and directly influences the n-6/n-3 ratio.

The n-6/n-3 ratio was lowest in the olive leaf–spent tea BSFL group, decreasing to 11.81 ± 0.65 compared with 14.49 ± 0.84 in the 10% standard full-fat BSFL group. Although this ratio remained higher than the ideal nutritional target generally recommended for human diets, the reduction observed in the olive leaf–spent tea group represents a meaningful improvement within the context of broiler breast meat. This result supports the idea that the lipid quality of BSFL meal can be partially improved through larval substrate manipulation. It also confirms that the effect of BSFL meal on meat lipids should not be interpreted solely through inclusion percentage, but also through the chemical quality of the insect biomass.

The PUFA/SFA ratio further supported the nutritional advantage of the olive leaf–spent tea BSFL meal. The lowest value was observed in the 10% standard full-fat BSFL group (0.69 ± 0.03), whereas the olive leaf–spent tea group showed a higher ratio (0.77 ± 0.04), close to the control value (0.78 ± 0.04). This indicates that the functional BSFL meal limited the saturated-fat enrichment induced by the standard full-fat BSFL meal. Similar diet-dependent changes in breast meat fatty acid composition have been reported in broilers fed BSFL meal or BSFL fat, confirming that insect-derived lipids can be reflected in the final meat lipid profile [15].

Atherogenicity and thrombogenicity indices confirmed the same trend. The atherogenicity index was highest in the 10% standard full-fat BSFL group (0.46 ± 0.04) and lower in the olive leaf–spent tea group (0.40 ± 0.03). Similarly, the thrombogenicity index decreased from 0.92 ± 0.06 in the 10% standard full-fat BSFL group to 0.78 ± 0.05 in the olive leaf–spent tea group. These indices integrate the relative contribution of hypercholesterolemic saturated fatty acids and protective unsaturated fatty acids. Therefore, the lower values observed in the olive leaf–spent tea group indicate a healthier lipid index profile than that of the standard full-fat BSFL meal at the same inclusion level.

The comparison between the two 10% full-fat BSFL treatments is central to the novelty of this study. Both diets contained the same inclusion level of full-fat BSFL meal, but the standard meal increased C12:0, C14:0, C16:0, total SFA, the atherogenicity index and the thrombogenicity index, whereas the olive leaf–spent tea meal increased C18:1 n-9, C18:2 n-6, C18:3 n-3 and n-3 PUFA, while reducing the n-6/n-3 ratio. This demonstrates that the lipid response was not driven only by the presence of BSFL meal in the diet, but also by the substrate-dependent quality of the larvae meal. Thus, substrate tailoring appears to be a relevant strategy for improving the nutritional expression of BSFL-derived lipids in broiler meat.

The defatted BSFL meal produced a fatty acid profile closer to the control than to the standard full-fat BSFL group. This was expected because lipid removal reduced the amount of BSFL fat entering the diet and limited the deposition of medium-chain saturated fatty acids in breast meat. The defatted group showed lower lauric and myristic acid contents than the 10% standard full-fat BSFL group and maintained relatively favorable PUFA/SFA, atherogenicity and thrombogenicity indices. However, defatting did not reproduce the higher oleic acid and a-linolenic acid values observed in the olive leaf–spent tea group. This distinction is important because defatted BSFL meal behaves mainly as a protein ingredient, whereas substrate-tailored full-fat BSFL meal may provide protein, lipids and functional compounds simultaneously.

From an oxidative-stability perspective, the olive leaf–spent tea BSFL group requires careful interpretation. Higher contents of unsaturated fatty acids, particularly PUFA and a-linolenic acid, can increase susceptibility to lipid oxidation during refrigerated storage. However, this treatment also originated from a BSFL meal with higher phenolic content, stronger antioxidant capacity and lower initial lipid oxidation. Therefore, the improved nutritional lipid profile of the olive leaf–spent tea group does not necessarily imply poorer oxidative stability. This point is directly evaluated in the following subsection through TBARS, protein carbonyls, redness stability and volatile oxidation markers. Overall, the results show that standard full-fat BSFL meal increased the deposition of medium-chain and saturated fatty acids in broiler breast meat, whereas olive leaf–spent tea BSFL meal attenuated this effect and improved several nutritional lipid indices. The lower n-6/n-3 ratio, lower thrombogenicity index and higher oleic and a-linolenic acid contents observed in the olive leaf–spent tea group support the concept of substrate-tailored BSFL meal as a functional ingredient capable of improving not only the sustainability and protein supply, but also the nutritional lipid quality of broiler meat.

### 3.5. Lipid Oxidation, Protein Oxidation and Color Stability During Refrigerated Storage

**Figure 1:**
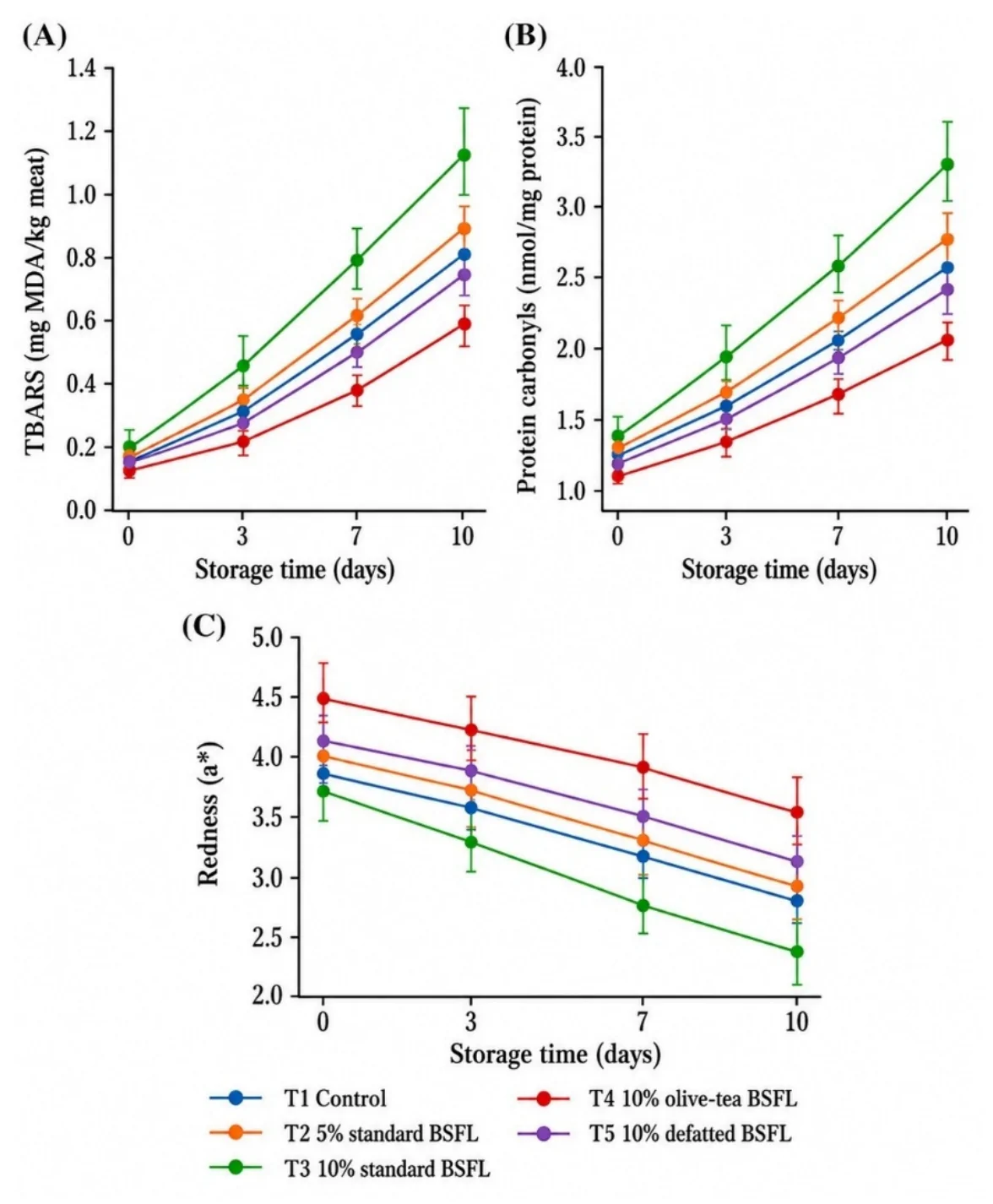
Effect of dietary BSFL meal type and inclusion level on broiler meat oxidative stability and color stability during refrigerated storage. Temporal changes in (**A**) lipid oxidation, expressed as TBARS values (mg MDA/kg meat), (**B**) protein oxidation, expressed as protein carbonyl content (nmol/mg protein), and (**C**) redness, expressed as a value, in broiler meat stored under refrigerated conditions for 0, 3, 7, and 10 days. Data are presented as mean ± SD. T1 = control diet without BSFL meal; T2 = diet containing 5% standard full-fat BSFL meal; T3 = diet containing 10% standard full-fat BSFL meal; T4 = diet containing 10% polyphenol-enriched full-fat BSFL meal produced from larvae reared on olive leaves and spent tea residues; T5 = diet containing 10% defatted standard BSFL meal. BSFL = black soldier fly larvae.

The evolution of TBARS, protein carbonyls, and redness (a*) during refrigerated storage is shown in Figure 1. Storage time was associated with progressive increases in lipid and protein oxidation and a concomitant decrease in redness, indicating gradual oxidative deterioration of broiler breast meat during refrigeration. These changes are consistent with the interrelated oxidation of unsaturated lipids, muscle proteins, and heme pigments during post-mortem storage. TBARS values increased in all treatments from day 0 to day 10, indicating progressive accumulation of secondary lipid oxidation products. The highest increase was observed in the 10% standard full-fat BSFL group (T3), from 0.22 to 1.12 mg MDA/kg meat, whereas the olive leaf–spent tea BSFL group (T4) showed the lowest increase, from 0.16 to 0.60 mg MDA/kg meat. Thus, at the common 10% full-fat BSFL inclusion level, meat from T4 showed lower lipid oxidation during refrigerated storage than meat from T3.

The greater TBARS accumulation in T3 may reflect differences in the oxidative characteristics of the dietary BSFL meals rather than in fatty-acid unsaturation alone. Meat lipid oxidation is influenced by several interacting factors, including fatty-acid composition, the initial oxidative status of dietary lipids, antioxidant availability, pro-oxidant compounds, and interactions between lipid and protein oxidation [25,56]. In the present study, the standard full-fat BSFL meal showed higher initial peroxide and TBARS values than the olive leaf–spent tea BSFL meal. This difference in initial oxidative status may have contributed to the subsequent differences observed in breast meat, although a direct causal relationship cannot be established.

The T4 response is particularly noteworthy because this treatment contained higher proportions of several unsaturated fatty acids than T3 while maintaining lower TBARS values during storage. Therefore, the greater unsaturation of T4 meat did not correspond to greater oxidative deterioration under the present experimental conditions. One possible explanation is that the higher antioxidant-related characteristics of the olive leaf–spent tea BSFL meal partially counteracted the susceptibility of unsaturated lipids to oxidation. Dietary olive leaf extract has previously been associated with improved antioxidant status and reduced malondialdehyde concentrations in broiler breast meat [57]. Tea residues also retain bioactive polyphenolic compounds with antioxidant potential [58], while plant-derived extracts, including tea-derived antioxidants, have been shown to retard lipid oxidation in poultry meat during chilled storage [56]. However, phenolic deposition or bioavailability in breast muscle was not directly determined in the present study. Consequently, the contribution of these compounds to meat’s oxidative stability remains a plausible interpretation rather than a demonstrated mechanism.

Protein carbonyl concentrations also increased during storage in all treatments, confirming progressive protein oxidation. T3 showed the greatest increase, from 1.36 to 3.35 nmol/mg protein between day 0 and day 10, whereas T4 increased from 1.12 to 2.16 nmol/mg protein. These results indicate lower accumulation of protein-oxidation products in T4 than in T3 during refrigerated storage. Protein carbonyl formation is widely used as an indicator of oxidative modification of muscle proteins, and lipid and protein oxidation may develop concomitantly during meat storage [25,54]. The parallel evolution of TBARS and protein carbonyls observed in the present study is therefore consistent with interconnected oxidative processes within the meat matrix.

The lower carbonyl accumulation observed in T4 is also consistent with the lower drip and cooking losses reported previously for this treatment. Oxidative modification of muscle proteins can alter protein structure and functionality and may consequently affect water retention [54,55]. Nevertheless, because muscle protein functionality was not directly characterized in the present study, the data do not demonstrate that reduced protein oxidation was the direct cause of the differences in drip or cooking loss.

Redness decreased progressively during refrigerated storage in all treatments. The greatest decline was observed in T3, where a* decreased from 3.75 at day 0 to 2.40 at day 10, whereas T4 decreased from 4.46 to 3.52 and maintained comparatively higher redness throughout storage. This pattern indicates greater preservation of the red color component in T4 under the present storage conditions. Oxidative processes involving lipids and muscle pigments are closely associated with meat discoloration, and plant-derived polyphenols have been investigated for their capacity to limit pigment oxidation and improve color stability [56,59]. The better maintenance of redness in T4 occurred alongside lower TBARS and protein carbonyl values. Because lipid, protein, and pigment oxidation are interconnected in meat systems, these parallel responses suggest lower overall oxidative deterioration in T4. However, the present measurements establish an association rather than a direct mechanistic relationship among these oxidation pathways.

The defatted BSFL group (T5) showed an intermediate response, with the TBARS and protein carbonyl values generally being lower than those of T3 but higher than those of T4, while redness was also intermediate between the two treatments. These observations suggest that defatting and substrate modification produced different oxidative responses. However, the experimental design does not permit the effects of lipid removal and substrate-derived characteristics to be completely separated mechanistically.

### 3.6. Volatile Oxidation Markers During Refrigerated Storage

**Figure 2:**
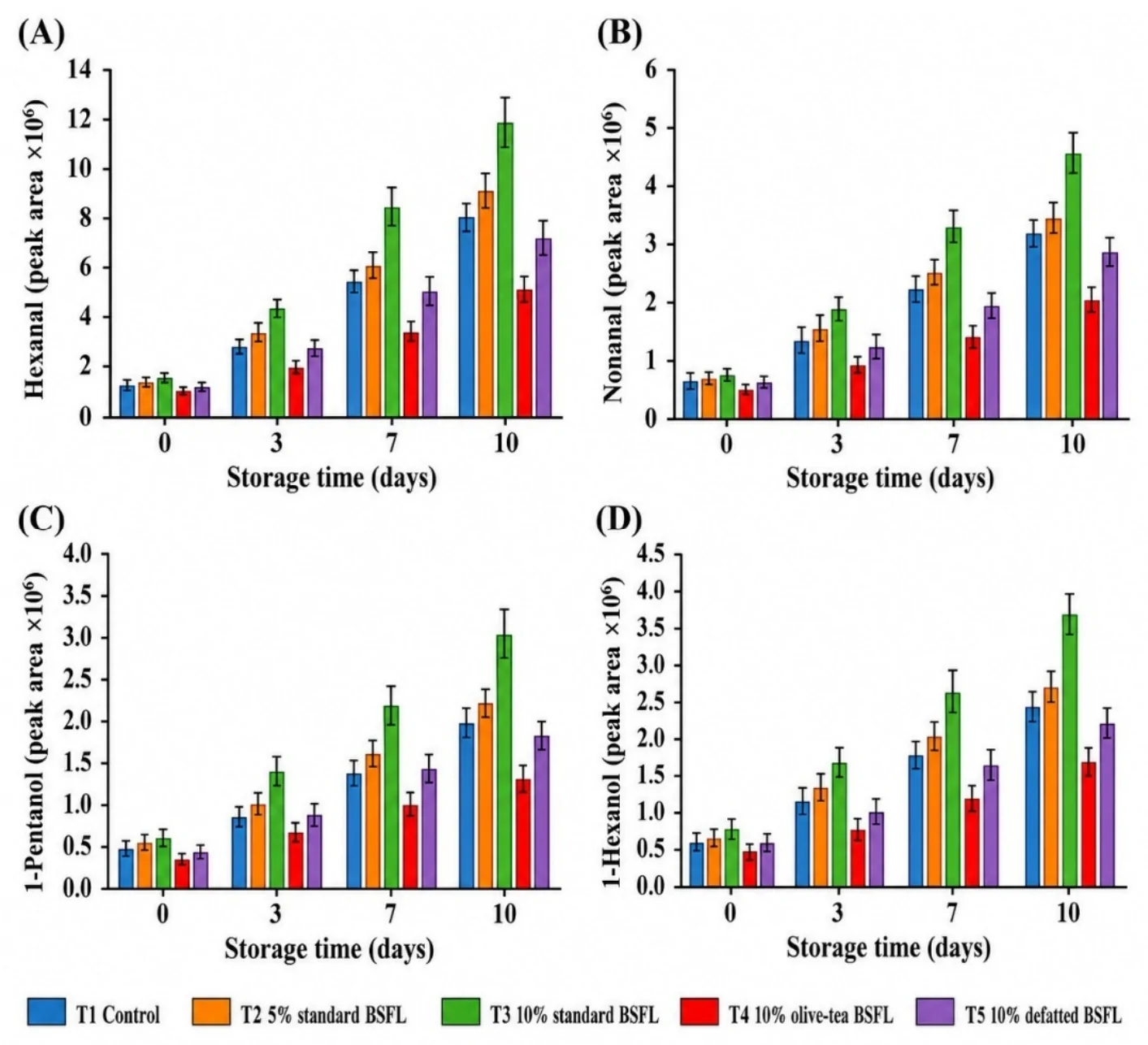
Effect of dietary BSFL meal type and inclusion level on volatile oxidation markers of broilers breast meat during refrigerated storage. Temporal changes in (**A**) hexanal, (**B**) nonanal, (**C**) 1-pentanol, and (**D**) 1-hexanol in broiler breast meat stored under refrigerated conditions for 0, 3, 7, and 10 days. These compounds were monitored as representative aldehyde and alcohol volatile markers associated with lipid oxidation and storage-related quality deterioration. Values are expressed as peak area × 105 and presented as mean ± SD. T1 = control diet without BSFL meal; T2 = diet containing 5% standard full-fat BSFL meal; T3 = diet containing 10% standard full-fat BSFL meal; T4 = diet containing 10% polyphenol-enriched full-fat BSFL meal produced from larvae reared on olive leaves and spent tea residues; T5 = diet containing 10% defatted standard BSFL meal. BSFL = black soldier fly larvae.

The selected volatile oxidation markers of breast meat are shown in Figure 2. Hexanal, nonanal, 1-pentanol and 1-hexanol increased progressively during refrigerated storage, confirming the formation of lipid-derived volatile compounds over time. This pattern agrees with the TBARS results and indicates that lipid oxidation was expressed not only through malondialdehyde formation, but also through the accumulation of volatile compounds associated with oxidative flavor deterioration. This is important because volatile aldehydes and alcohols are more directly related to aroma deterioration than global oxidation indices alone.

Hexanal showed the clearest treatment effect. At day 10, the 10% standard full-fat BSFL group showed the highest hexanal abundance, whereas the olive leaf–spent tea BSFL group showed the lowest value. This result is particularly relevant because hexanal is mainly generated from the oxidation of n-6 polyunsaturated fatty acids, especially linoleic acid, and is widely used as a marker of rancid odor and oxidative off-flavor in meat systems. Therefore, the high hexanal accumulation in the standard full-fat BSFL group confirms the stronger lipid oxidation already observed through TBARS.

The lower hexanal level in the olive leaf–spent tea BSFL group provides strong evidence of improved oxidative flavor stability. This treatment had a more unsaturated fatty acid profile than that of the 10% standard full-fat BSFL group, which could theoretically increase oxidation susceptibility. However, it produced less hexanal during storage. This suggests that the antioxidant-related properties of the olive leaf–spent tea BSFL meal were sufficient to counterbalance the higher oxidizability of its unsaturated lipid fraction. Thus, the volatile profile confirms that lipid nutritional improvement did not occur at the expense of oxidative shelf-life.

Nonanal followed a similar trend, indicating broader oxidative degradation of unsaturated fatty acids. Unlike hexanal, which is strongly associated with linoleic acid oxidation, nonanal is commonly linked with the oxidation of oleic acid and other unsaturated lipid fractions. Its higher accumulation in the standard full-fat BSFL group suggests that oxidative deterioration was not limited to a single fatty acid pathway, but involved wider lipid degradation during refrigerated storage. Conversely, the lower nonanal abundance in the olive leaf–spent tea group supports the protective role of substrate functionalization against multiple volatile oxidation routes.

The alcohols 1-pentanol and 1-hexanol also increased during storage, with the highest values observed in the 10% standard full-fat BSFL group and the lowest values in the olive leaf–spent tea BSFL group. These compounds can arise from the degradation or transformation of lipid hydroperoxides and aldehydes. Although alcohols are generally less reactive than aldehydes, their accumulation remains useful because it reflects advanced lipid oxidation and contributes to the global volatile profile of stored meat. Their lower abundance in the olive leaf–spent tea group therefore confirms a slower progression of the formation of oxidation-derived volatiles.

The comparison between the two 10% full-fat BSFL treatments is central to this subsection. Both groups received the same inclusion level of full-fat BSFL meal, but the standard full-fat meal produced the highest levels of hexanal, nonanal, 1-pentanol and 1-hexanol, whereas the olive leaf–spent tea meal consistently limited their accumulation. This confirms that volatile formation was not determined only by BSFL inclusion level. Instead, it depended strongly on the chemical quality, initial oxidative status and antioxidant-related profile of the larvae meal.

The lower volatile production in the olive leaf–spent tea group is consistent with the previous oxidative-stability results. This treatment showed lower TBARS, lower protein carbonyls and better redness stability during refrigerated storage. These convergent responses indicate that lipid oxidation, protein oxidation, pigment oxidation and volatile formation were part of the same deterioration process. In this context, the volatile data provide an aroma-relevant confirmation of the chemical protection observed through TBARS and carbonyl measurements.

The protective effect observed in the olive leaf–spent tea group may be attributed to the antioxidant potential of the larval substrate. Olive leaf phenolics, particularly oleuropein, hydroxytyrosol and tyrosol, and tea-derived catechins, can delay lipid oxidation through radical scavenging, metal chelation and interruption of oxidative chain reactions. By limiting lipid hydroperoxide formation and decomposition, these compounds may reduce the generation of aldehydes and alcohols during refrigerated storage. However, because phenolic residues were not directly quantified in meat, this mechanism should be interpreted as a plausible explanation supported by the antioxidant profile of the meal and the volatile-response pattern.

The defatted BSFL group showed an intermediate volatile profile. Volatile oxidation markers were generally lower than in the 10% standard full-fat BSFL group but higher than in the olive leaf–spent tea group. This indicates that defatting reduced lipid-related oxidative pressure by limiting the transfer of BSFL-derived fat into the diet. Nevertheless, defatting did not provide the same protection as substrate enrichment with antioxidant-rich by-products. Therefore, lipid removal reduced the substrate available for oxidation, whereas olive leaf–spent tea functionalization appeared to actively limit oxidation progression. Overall, the volatile profile confirms and strengthens the TBARS, protein carbonyl and color-stability findings. Standard full-fat BSFL meal at 10% inclusion accelerated the accumulation of lipid-derived aldehydes and alcohols during refrigerated storage, especially hexanal. In contrast, the olive leaf–spent tea BSFL meal markedly reduced volatile oxidation-marker formation, despite its more favorable unsaturated fatty acid profile. These findings demonstrate that substrate-tailored BSFL meal can improve not only the chemical oxidative stability of broiler breast meat but also its potential flavor stability during refrigerated storage.

### 3.7. Integrated Correlation and PCA

**Figure 3:**
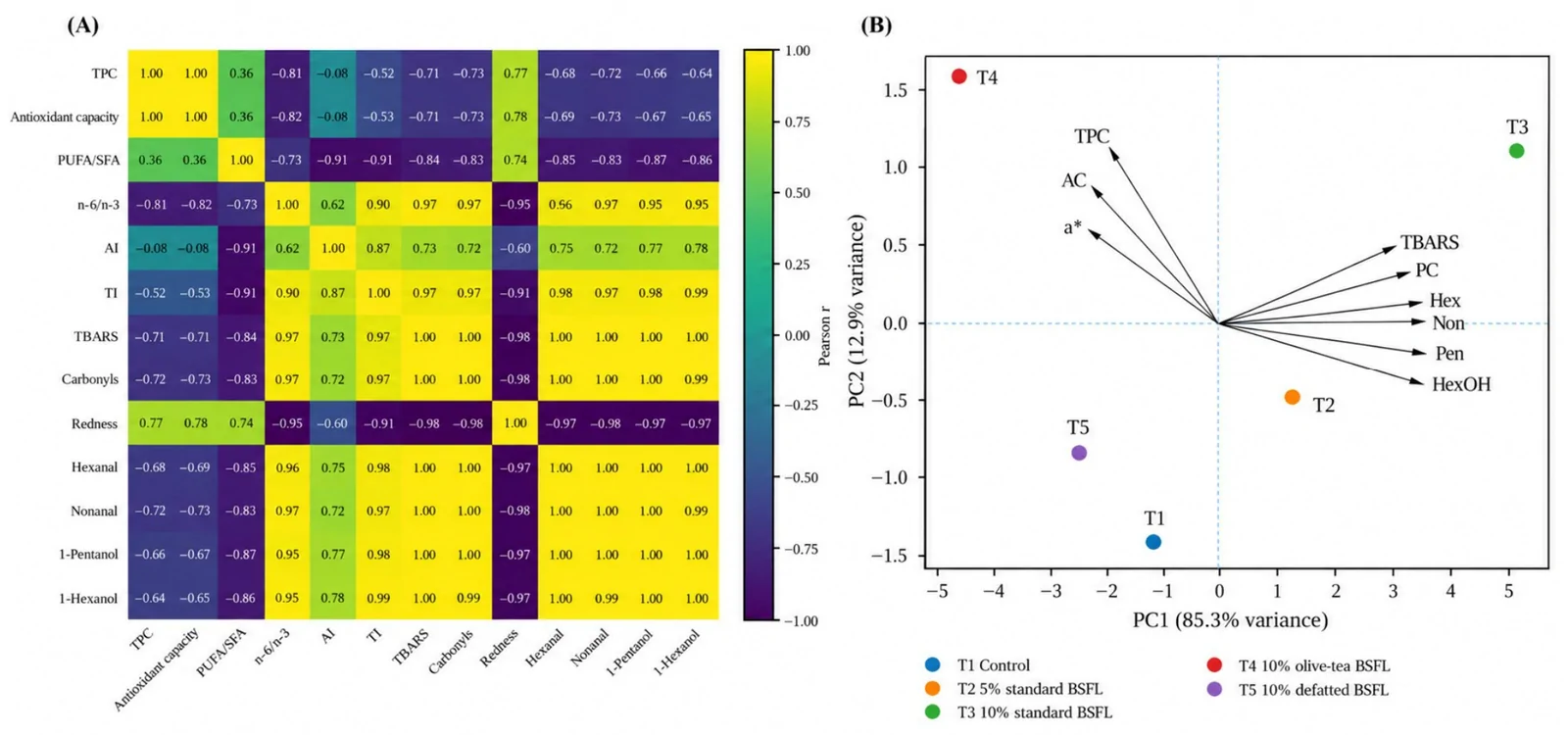
Integrated correlation and principal component analysis of antioxidant-related traits, oxidative stability, color, and volatile markers in broiler breast meat. (**A**) Pearson’s correlation heatmap showing the relationships among total phenolic content (TPC), antioxidant capacity (AC), PUFA/SFA ratio, redness (a*), oxidative stability indices [TBARS and protein carbonyls (PC)], and volatile compounds (hexanal, nonanal, 1-pentanol, and 1-hexanol) in broiler breast meat. (**B**) Principal component analysis (PCA) biplot illustrating the distribution of dietary treatment groups and their associations with the measured variables. PC1 and PC2 explained 85.3% and 12.9% of the total variance, respectively. T1 = control diet without BSFL meal; T2 = diet containing 5% standard full-fat BSFL meal; T3 = diet containing 10% standard full-fat BSFL meal; T4 = diet containing 10% polyphenol-enriched full-fat BSFL meal produced from larvae reared on olive leaves and spent tea residues; T5 = diet containing 10% defatted standard BSFL meal. BSFL = black soldier fly larvae.

The correlation heatmap and PCA biplot are shown in Figure 3. This integrated analysis was performed to connect the main chemical responses observed throughout the study and to determine whether the different BSFL meals produced distinct meat-quality signatures. The heatmap revealed strong positive associations among TBARS, protein carbonyls, hexanal, nonanal, 1-pentanol and 1-hexanol. This confirms that lipid oxidation, protein oxidation and volatile formation occurred concomitantly during refrigerated storage. Chemically, this relationship is coherent because lipid hydroperoxides decompose into aldehydes and alcohols, while lipid-derived radicals and carbonyl compounds can promote oxidative modifications of muscle proteins.

Redness was negatively associated with TBARS, protein carbonyls and volatile oxidation markers. This indicates that oxidative deterioration was linked to color loss during storage. The relationship is biologically meaningful because lipid oxidation, protein oxidation and pigment oxidation are not independent events in meat systems. As oxidative reactions progress, lipid-derived radicals and aldehydes can accelerate myoglobin oxidation and reduce the stability of the red pigment state. Therefore, the negative association between redness and oxidation markers supports the interpretation that improved color stability in the olive leaf–spent tea BSFL group resulted from lower oxidative pressure in the breast meat matrix.

Antioxidant-related variables, particularly total phenolic content and antioxidant capacity, were negatively associated with oxidation markers and positively associated with redness. This confirms that the antioxidant enrichment of the olive leaf–spent tea BSFL meal was aligned with lower lipid oxidation, lower protein carbonylation, reduced volatile formation and better color preservation. These relationships strengthen the mechanistic interpretation proposed in the previous subsections: antioxidant-rich substrates can improve the downstream oxidative stability of broiler meat by modifying the chemical and functional quality of BSFL meal.

The PCA biplot further supported the separation of treatments according to oxidative and antioxidant profiles. The 10% standard full-fat BSFL group was associated with TBARS, protein carbonyls, hexanal, nonanal, 1-pentanol and 1-hexanol, confirming its higher oxidative instability. In contrast, the olive leaf–spent tea BSFL group was positioned in the opposite direction and was associated with antioxidant-related variables and redness stability. This separation is particularly important because both treatments contained 10% full-fat BSFL meal. Therefore, the PCA confirms that the meat-quality response was not determined by inclusion level alone, but by the chemical quality, oxidative status and antioxidant-related profile of the larvae meal.

The position of the defatted BSFL group was intermediate. This suggests that lipid removal reduced oxidative pressure compared with the standard full-fat BSFL meal, probably by limiting the dietary transfer of BSFL-derived lipids and medium-chain saturated fatty acids. However, the defatted meal did not provide the same protection as the olive leaf–spent tea BSFL meal. This distinction is important because it shows that defatting and substrate functionalization do not have the same biological meaning. Defatting mainly reduces lipid exposure, whereas substrate tailoring appears to improve the antioxidant quality of the ingredient and its downstream effect on meat stability.

The PCA also provides a useful global interpretation of the study. The control and 5% standard BSFL groups were less strongly associated with oxidation markers than the 10% standard full-fat BSFL group, indicating that the oxidative response increased when standard full-fat BSFL meal was included at the higher level. However, this effect was not observed when the same 10% inclusion level was supplied through olive leaf–spent tea BSFL meal. This confirms that high inclusion of full-fat BSFL meal is not necessarily detrimental, provided that the larvae meal has a favorable oxidative and functional profile. Overall, the correlation and PCA analyses integrate the main findings of the study into a coherent mechanism. Standard full-fat BSFL meal at 10% inclusion was associated with higher saturated lipid deposition, greater lipid oxidation, protein carbonylation, volatile formation and redness loss. In contrast, olive leaf–spent tea BSFL meal was associated with higher antioxidant-related traits, better lipid nutritional quality, lower oxidative deterioration and improved color stability. These multivariate results support the central conclusion that substrate-tailored BSFL meal can act as a functional feed ingredient capable of improving broiler breast meat quality and refrigerated oxidative stability.

## 4. Conclusions

The present study indicates that the nutritional, oxidative, and functional characteristics of BSFL meal can vary according to larval rearing substrate and post-processing treatment, with associated differences in broiler performance and breast meat quality. Compared with the standard full-fat BSFL meal, the olive leaf–spent tea BSFL meal showed higher phenolic content, greater antioxidant capacity, lower initial lipid oxidation, and a slightly more favorable unsaturated fatty acid profile, with no evidence of increased heavy-metal contamination under the conditions evaluated. These findings highlight that BSFL meal should not be considered a chemically uniform feed ingredient, because its composition and functional characteristics can depend on production conditions.

At the common 10% inclusion level, birds receiving the olive leaf–spent tea full-fat BSFL meal (T4) had higher final body weight and body weight gain and a lower feed conversion ratio than those receiving the standard full-fat BSFL meal (T3), whereas feed intake, mortality, and viability were not significantly different. At the meat level, T4 was associated with better redness preservation and lower drip and cooking losses than T3. These responses should, however, be interpreted as treatment-associated differences rather than as direct effects of dietary polyphenols, because phenolic transfer to muscle was not measured. The breast meat fatty acid profile also differed between the two 10% full-fat BSFL treatments. Compared with T3, T4 showed higher oleic and α-linolenic acid contents, a lower n-6/n-3 ratio, and a lower thrombogenicity index. These results suggest that the fatty acid response to BSFL-based diets depends not only on inclusion level but also on the compositional characteristics of the insect meal.

During refrigerated storage, breast meat from T4 showed lower accumulation of TBARS, protein carbonyls, and volatile oxidation markers, together with better preservation of redness compared with T3. Correlation and PCA analyses supported the association of T4 with antioxidant-related characteristics and lower oxidative deterioration, whereas T3 was more closely associated with oxidation and volatile-formation markers. These multivariate associations should not be interpreted as evidence of a direct causal pathway. In particular, although the antioxidant-related properties of the olive leaf–spent tea BSFL meal may have contributed to the observed response, the transfer and bioavailability of individual phenolic compounds in breast muscle were not determined.

The interpretation of these findings is also constrained by the unbalanced treatment structure, because standard BSFL meal was evaluated at 5% and 10%, whereas the olive leaf–spent tea and defatted meals were evaluated only at 10%. In addition, inferences about growth performance were based on four replicate pens per treatment, and the compositional characterization of each BSFL meal was based on pooled material with analytical rather than independent biological replication. Consequently, dose-independent effects and causal effects of polyphenol enrichment cannot be established from the present design. Overall, the results support further evaluation of olive leaf and spent tea residues as alternative rearing substrates for producing BSFL meal with potentially favorable nutritional and oxidative characteristics. Under the present experimental conditions, the olive leaf–spent tea BSFL meal at 10% inclusion was associated with a more favorable breast meat lipid profile and improved oxidative stability compared with the standard full-fat BSFL meal at the same inclusion level. Future studies should validate these findings using balanced dose–response designs, larger numbers of independent production and pen replicates, direct assessment of phenolic transfer and antioxidant mechanisms, and sensory and microbiological evaluations before conclusions regarding commercial shelf-life or consumer relevance can be made.

## Figures and Tables

**Table 1:** Ingredient composition and calculated nutrient levels of the experimental broiler diets during the starter, grower, and finisher phases.

A. Starter Phase (Days 1–10)					
Ingredient, %	T1	T2	T3	T4	T5
Maize	57.65	58.7	59.75	59.55	62.2
Soybean meal	35.75	32.2	28.65	28.85	24.1
Standard full-fat BSFL meal	0	5	10	0	0
Olive leaf–spent tea full-fat BSFL meal	0	0	0	10	0
Defatted standard BSFL meal	0	0	0	0	10
Vegetable oil	2.75	1.7	0.65	0.85	2.85
Dicalcium phosphate	1.2	1.05	0.9	0.9	0.9
Limestone	1.2	1	0.9	0.9	0.9
Sodium chloride	0.5	0.5	0.5	0.5	0.5
DL-Methionine	0.38	0.35	0.33	0.33	0.35
L-Lysine HCl	0.22	0.15	0.1	0.1	0.15
L-Threonine	0.08	0.05	0.03	0.03	0.05
Choline chloride	0.05	0.05	0.05	0.05	0.05
Vitamin–mineral premix	0.22	0.22	0.22	0.22	0.22
Metabolizable energy, kcal/kg	3000	3000	3000	3000	3000
Crude protein, %	22	22	22	22	22
Calcium, %	0.95	0.95	0.95	0.95	0.95
Available phosphorus, %	0.45	0.45	0.45	0.45	0.45
Digestible lysine, %	1.22	1.22	1.22	1.22	1.22
Digestible Met + Cys, %	0.95	0.95	0.95	0.95	0.95
Grower phase (days 11–24)					
Maize	61.55	62.55	63.55	63.35	66
Soybean meal	31.3	27.75	24.2	24.4	19.65
Standard full-fat BSFL meal	0	5	10	0	0
Olive leaf–spent tea full-fat BSFL meal	0	0	0	10	0
Defatted standard BSFL meal	0	0	0	0	10
Vegetable oil	3.2	2.15	1.1	1.3	3.3
Dicalcium phosphate	1.05	0.9	0.75	0.75	0.75
Limestone	1.15	0.95	0.85	0.85	0.85
Sodium chloride	0.45	0.45	0.45	0.45	0.45
DL-Methionine	0.3	0.28	0.25	0.25	0.28
L-Lysine HCl	0.2	0.13	0.08	0.08	0.13
L-Threonine	0.05	0.03	0	0	0.03
Choline chloride	0.05	0.05	0.05	0.05	0.05
Vitamin–mineral premix	0.2	0.2	0.2	0.2	0.2
Metabolizable energy, kcal/kg	3100	3100	3100	3100	3100
Crude protein, %	20.5	20.5	20.5	20.5	20.5
Calcium, %	0.88	0.88	0.88	0.88	0.88
Available phosphorus, %	0.42	0.42	0.42	0.42	0.42
Digestible lysine, %	1.12	1.12	1.12	1.12	1.12
Digestible Met + Cys, %	0.85	0.85	0.85	0.85	0.85
Finisher phase (days 25–42)					
Maize	66.15	67.15	68.15	67.95	70.6
Soybean meal	26.65	23.1	19.55	19.75	15
Standard full-fat BSFL meal	0	5	10	0	0
Olive leaf–spent tea full-fat BSFL meal	0	0	0	10	0
Defatted standard BSFL meal	0	0	0	0	10
Vegetable oil	3.65	2.6	1.55	1.75	3.75
Dicalcium phosphate	0.9	0.75	0.65	0.65	0.65
Limestone	1.05	0.9	0.8	0.8	0.8
Sodium chloride	0.42	0.42	0.42	0.42	0.42
DL-Methionine	0.25	0.22	0.2	0.2	0.22
L-Lysine HCl	0.18	0.11	0.06	0.06	0.11
L-Threonine	0.04	0.02	0	0	0.02
Choline chloride	0.05	0.05	0.05	0.05	0.05
Vitamin–mineral premix	0.2	0.2	0.2	0.2	0.2
Metabolizable energy, kcal/kg	3200	3200	3200	3200	3200
Crude protein, %	19	19	19	19	19
Calcium, %	0.8	0.8	0.8	0.8	0.8
Available phosphorus, %	0.38	0.38	0.38	0.38	0.38
Digestible lysine, %	1.02	1.02	1.02	1.02	1.02
Digestible Met + Cys, %	0.75	0.75	0.75	0.75	0.75

T1 = control diet without BSFL meal; T2 = diet containing 5% standard full-fat BSFL meal; T3 = diet containing 10% standard full-fat BSFL meal; T4 = diet containing 10% polyphenol-enriched full-fat BSFL meal produced from larvae reared on olive leaves and spent tea residues; T5 = diet containing 10% defatted standard BSFL meal. BSFL = black soldier fly larvae.

**Table 2:** Chemical composition, lipid profile, antioxidant-related traits and safety indicators of black soldier fly larvae meal.

Parameter	Standard Full-Fat BSFL Meal	Olive Leaf + Spent Tea BSFL Meal	Defatted BSFL Meal	SEM	*p*-Value
**Proximate composition**					
Dry matter, %	93.48 ^ab^	92.91 ^b^	94.26 ^a^	0.21	0.032
Crude protein, % DM	41.20 ^b^	40.35 ^b^	58.76 ^a^	1.15	<0.001
Corrected protein, % DM	31.37 ^b^	30.72 ^b^	44.85 ^a^	0.93	<0.001
Crude fat, % DM	34.65 ^a^	31.82 ^b^	8.74 ^c^	0.84	<0.001
Ash, % DM	8.42 ^c^	9.31 ^b^	11.05 ^a^	0.26	<0.001
Chitin, % DM	7.63 ^b^	7.95 ^b^	10.18 ^a^	0.31	<0.001
**Antioxidant-related traits**					
Total phenolic content, mg GAE/g DM	1.82 ^b^	6.74 ^a^	1.96 ^b^	0.23	<0.001
DPPH inhibition, %	22.40 ^b^	54.80 ^a^	24.10 ^b^	1.7	<0.001
ABTS, µmol TE/g DM	15.60 ^b^	38.20 ^a^	16.90 ^b^	1.2	<0.001
Peroxide value, meq O_2_/kg lipid	2.38 ^a^	1.46 ^b^	2.21 ^a^	0.14	0.004
TBARS, mg MDA/kg meal	0.39 ^a^	0.24 ^b^	0.36 ^a^	0.03	0.011
**Fatty acid groups**					
SFA, % total FA	72.85 ^a^	68.10 ^b^	73.42 ^a^	0.88	0.006
MUFA, % total FA	17.35 ^b^	20.80 ^a^	17.10 ^b^	0.59	0.003
PUFA, % total FA	9.80 ^b^	11.10 ^a^	9.48 ^b^	0.31	0.018
PUFA/SFA ratio	0.13 ^b^	0.16 ^a^	0.13 ^b^	0.01	0.021
n-6/n-3 ratio	15.80 ^a^	12.40 ^b^	15.60 ^a^	0.72	0.015
**Major fatty acids**					
C12:0, lauric acid	43.60 ^a^	39.85 ^b^	44.10 ^a^	0.81	0.009
C14:0, myristic acid	8.95 ^a^	8.10 ^b^	9.05 ^a^	0.2	0.012
C16:0, palmitic acid	14.70 ^a^	14.05 ^a^	14.56 ^a^	0.35	0.421
C18:0, stearic acid	5.20 ^a^	5.08 ^a^	5.32 ^a^	0.16	0.612
C16:1 n-7, palmitoleic acid	2.10 ^b^	2.55 ^a^	2.06 ^b^	0.09	0.007
C18:1 n-9, oleic acid	14.20 ^b^	17.10 ^a^	13.96 ^b^	0.48	0.002
C18:2 n-6, linoleic acid	8.70 ^b^	9.55 ^a^	8.42 ^b^	0.2	0.01
C18:3 n-3, α-linolenic acid	0.55 ^b^	0.77 ^a^	0.54 ^b^	0.04	0.008
**Mineral composition**					
Calcium, g/kg DM	27.60 ^a^	29.85 ^a^	31.20 ^a^	1.05	0.084
Phosphorus, g/kg DM	8.90 ^b^	9.35 ^b^	11.10 ^a^	0.36	0.004
Magnesium, g/kg DM	3.10 ^b^	3.86 ^a^	3.65 ^a^	0.14	0.006
Iron, mg/kg DM	184.0 ^b^	226.5 ^a^	205.8 ^ab^	9.6	0.031
Zinc, mg/kg DM	112.4 ^a^	118.7 ^a^	121.6 ^a^	5.2	0.438
Copper, mg/kg DM	17.6 ^b^	22.4 ^a^	20.8 ^ab^	1.1	0.028
Manganese, mg/kg DM	38.5 ^b^	52.6 ^a^	48.2 ^a^	2.3	0.003
**Heavy metals**					
Lead, mg/kg DM	0.082 ^a^	0.095 ^a^	0.089 ^a^	0.006	0.338
Cadmium, mg/kg DM	0.041 ^a^	0.052 ^a^	0.046 ^a^	0.004	0.196
Arsenic, mg/kg DM	0.030 ^a^	0.036 ^a^	0.034 ^a^	0.003	0.441
Mercury, mg/kg DM	0.006 ^a^	0.007 ^a^	0.006 ^a^	0.001	0.728

Values are presented as means, with SD representing the pooled standard error of the mean. Within each row, different superscript letters indicate significant differences among BSFL meal types (*p* < 0.05); rows sharing the same letter are not significantly different. DM = dry matter; GAE = gallic acid equivalents; TE = Trolox equivalents; MDA = malondialdehyde; FA = fatty acids; SFA = saturated fatty acids; MUFA = monounsaturated fatty acids; PUFA = polyunsaturated fatty acids; ABTS = 2,2′-azino-bis(3-ethylbenzothiazoline-6-sulfonic acid); DPPH = 2,2-diphenyl-1-picrylhydrazyl.

**Table 3:** Effect of dietary BSFL meal type and inclusion level on broiler growth performance.

Parameter	T1	T2	T3	T4	T5	*p*-Value
Initial body weight, g	42.1 ± 0.4 ^a^	42.0 ± 0.4 ^a^	42.2 ± 0.4 ^a^	42.1 ± 0.4 ^a^	42.0 ± 0.4 ^a^	0.914
Final body weight, g	2528.4 ± 37.2 ^ab^	2554.7 ± 37.2 ^ab^	2511.6 ± 37.2 ^b^	2592.8 ± 37.2 ^a^	2536.3 ± 37.2 ^ab^	0.047
Body weight gain, g	2486.3 ± 36.8 ^ab^	2512.7 ± 36.8 ^ab^	2469.4 ± 36.8 ^b^	2550.7 ± 36.8 ^a^	2494.3 ± 36.8 ^ab^	0.045
Average daily gain, g/day	59.20 ± 0.88 ^ab^	59.83 ± 0.88 ^ab^	58.80 ± 0.88 ^b^	60.73 ± 0.88 ^a^	59.39 ± 0.88 ^ab^	0.045
Average daily feed intake, g/day	93.56 ± 1.22 ^a^	93.84 ± 1.22 ^a^	92.95 ± 1.22 ^a^	93.16 ± 1.22 ^a^	92.88 ± 1.22 ^a^	0.621
Feed conversion ratio	1.58 ± 0.02 ^ab^	1.57 ± 0.02 ^ab^	1.58 ± 0.02 ^a^	1.53 ± 0.02 ^b^	1.56 ± 0.02 ^ab^	0.038
Mortality, %	2.50 ± 2.24 ^a^	2.50 ± 2.24 ^a^	5.00 ± 2.24 ^a^	2.50 ± 2.24 ^a^	2.50 ± 2.24 ^a^	0.774
Viability, %	97.50 ± 2.24 ^a^	97.50 ± 2.24 ^a^	95.00 ± 2.24 ^a^	97.50 ± 2.24 ^a^	97.50 ± 2.24 ^a^	0.774

Values are presented as mean ± standard deviation (SD). For growth-performance variables, the pen was considered the experimental unit (*n* = 4 pens per treatment). Within each row, different superscript letters indicate significant differences among dietary treatments (*p* < 0.05); values sharing at least one superscript letter are not significantly different. T1 = control diet without BSFL meal; T2 = diet containing 5% standard full-fat BSFL meal; T3 = diet containing 10% standard full-fat BSFL meal; T4 = diet containing 10% polyphenol-enriched full-fat BSFL meal produced from larvae reared on olive leaves and spent tea residues; T5 = diet containing 10% defatted standard BSFL meal. BSFL = black soldier fly larvae. ADG = average daily gain; ADFI = average daily feed intake; FCR = feed conversion ratio.

**Table 4:** Effect of dietary BSFL meal type and inclusion level on the physicochemical quality and proximate composition of broiler breast and thigh meat.

Parameter	T1	T2	T3	T4	T5	*p*-Value
**Breast meat physicochemical traits**						
pH 24 h	5.82 ± 0.05 ^a^	5.83 ± 0.04 ^a^	5.85 ± 0.05 ^a^	5.81 ± 0.04 ^a^	5.82 ± 0.05 ^a^	0.286
L*—lightness	54.20 ± 1.28 ^a^	53.85 ± 1.21 ^a^	54.76 ± 1.35 ^a^	52.94 ± 1.18 ^b^	53.62 ± 1.25 ^ab^	0.041
a*—redness	3.92 ± 0.32 ^b^	4.08 ± 0.30 ^ab^	3.75 ± 0.34 ^b^	4.46 ± 0.29 ^a^	4.12 ± 0.31 ^ab^	0.018
b*—yellowness	8.14 ± 0.48 ^a^	8.25 ± 0.50 ^a^	8.42 ± 0.53 ^a^	7.86 ± 0.45 ^a^	8.05 ± 0.47 ^a^	0.174
Drip loss, %	2.36 ± 0.28 ^ab^	2.28 ± 0.25 ^ab^	2.55 ± 0.31 ^a^	2.02 ± 0.22 ^b^	2.21 ± 0.26 ^ab^	0.032
Cooking loss, %	23.84 ± 1.42 ^ab^	23.20 ± 1.35 ^ab^	24.68 ± 1.50 ^a^	22.15 ± 1.21 ^b^	23.06 ± 1.32 ^ab^	0.029
**Breast meat proximate composition**						
Moisture, g/100 g	74.26 ± 0.72 ^a^	74.08 ± 0.70 ^a^	74.58 ± 0.76 ^a^	73.82 ± 0.68 ^a^	74.10 ± 0.71 ^a^	0.263
Dry matter, g/100 g	25.74 ± 0.72 ^a^	25.92 ± 0.70 ^a^	25.42 ± 0.76 ^a^	26.18 ± 0.68 ^a^	25.90 ± 0.71 ^a^	0.263
Crude protein, g/100 g	22.85 ± 0.48 ^ab^	23.02 ± 0.46 ^ab^	22.60 ± 0.52 ^b^	23.48 ± 0.44 ^a^	23.18 ± 0.45 ^ab^	0.036
Intramuscular lipid, g/100 g	1.56 ± 0.18 ^ab^	1.52 ± 0.17 ^ab^	1.70 ± 0.20 ^a^	1.34 ± 0.15 ^b^	1.45 ± 0.16 ^ab^	0.027
Ash, g/100 g	1.12 ± 0.07 ^a^	1.14 ± 0.06 ^a^	1.10 ± 0.07 ^a^	1.16 ± 0.06 ^a^	1.15 ± 0.06 ^a^	0.391
**Thigh meat proximate composition**						
Moisture, g/100 g	73.08 ± 0.81 ^a^	72.96 ± 0.78 ^a^	73.20 ± 0.84 ^a^	72.62 ± 0.76 ^a^	72.85 ± 0.79 ^a^	0.417
Dry matter, g/100 g	26.92 ± 0.81 ^a^	27.04 ± 0.78 ^a^	26.80 ± 0.84 ^a^	27.38 ± 0.76 ^a^	27.15 ± 0.79 ^a^	0.417
Crude protein, g/100 g	20.86 ± 0.54 ^ab^	21.05 ± 0.50 ^ab^	20.58 ± 0.57 ^b^	21.42 ± 0.49 ^a^	21.16 ± 0.51 ^ab^	0.044
Intramuscular lipid, g/100 g	4.25 ± 0.38 ^ab^	4.18 ± 0.36 ^ab^	4.46 ± 0.41 ^a^	3.88 ± 0.34 ^b^	4.05 ± 0.35 ^ab^	0.039
Ash, g/100 g	1.06 ± 0.06 ^a^	1.08 ± 0.05 ^a^	1.05 ± 0.06 ^a^	1.10 ± 0.05 ^a^	1.09 ± 0.05 ^a^	0.352

Values are presented as mean ± standard deviation (SD). For meat-quality measurements, the two birds sampled from each pen were considered subsamples, with the pen retained as the experimental unit (*n* = 4 pens per treatment). Within each row, different superscript letters indicate significant differences among dietary treatments (*p* < 0.05); means sharing at least one superscript letter are not significantly different. Superscript letters are not shown when the overall treatment effect is not significant. T1 = control diet without BSFL meal; T2 = diet containing 5% standard full-fat BSFL meal; T3 = diet containing 10% standard full-fat BSFL meal; T4 = diet containing 10% polyphenol-enriched full-fat BSFL meal produced from larvae reared on olive leaves and spent tea residues; T5 = diet containing 10% defatted standard BSFL meal. BSFL = black soldier fly larvae; L* = lightness; a* = redness; b* = yellowness.

**Table 5:** The effect of dietary BSFL meal type and inclusion level on the fatty acid profile and lipid nutritional indices of broiler breast meat.

Parameter	T1	T2	T3	T4	**T5**	***p*** **-Value**
**Major fatty acids, % total FA**						
C12:0, lauric acid	0.42 ± 0.05 ^e^	1.35 ± 0.12 ^c^	2.24 ± 0.18 ^a^	1.80 ± 0.15 ^b^	0.75 ± 0.08 ^d^	<0.001
C14:0, myristic acid	0.64 ± 0.06 ^c^	0.82 ± 0.07 ^b^	1.04 ± 0.09 ^a^	0.88 ± 0.08 ^b^	0.70 ± 0.06 ^c^	<0.001
C16:0, palmitic acid	22.60 ± 0.74 ^b^	22.95 ± 0.70 ^b^	23.80 ± 0.82 ^a^	22.10 ± 0.68 ^b^	22.40 ± 0.71 ^b^	0.018
C18:0, stearic acid	7.10 ± 0.34 ^a^	7.00 ± 0.31 ^a^	7.20 ± 0.36 ^a^	6.85 ± 0.30 ^a^	6.95 ± 0.33 ^a^	0.284
C16:1 n-7, palmitoleic acid	3.25 ± 0.18 ^ab^	3.18 ± 0.17 ^ab^	3.04 ± 0.16 ^b^	3.42 ± 0.19 ^a^	3.31 ± 0.18 ^ab^	0.047
C18:1 n-9, oleic acid	39.05 ± 1.12 ^ab^	38.62 ± 1.05 ^ab^	37.48 ± 1.10 ^b^	40.52 ± 1.08 ^a^	39.40 ± 1.00 ^ab^	0.021
C18:2 n-6, linoleic acid	19.80 ± 0.82 ^ab^	19.42 ± 0.78 ^ab^	18.70 ± 0.80 ^b^	20.44 ± 0.75 ^a^	20.08 ± 0.79 ^ab^	0.038
C18:3 n-3, α-linolenic acid	1.50 ± 0.10 ^b^	1.40 ± 0.09 ^b^	1.29 ± 0.08 ^b^	1.72 ± 0.11 ^a^	1.56 ± 0.10 ^ab^	0.006
Other identified fatty acids	5.64 ± 0.30 ^a^	5.26 ± 0.28 ^ab^	5.21 ± 0.27 ^ab^	2.27 ± 0.20 ^c^	4.85 ± 0.25 ^b^	<0.001
**Fatty acid groups, % total FA**						
SFA	31.40 ± 0.95 ^c^	32.60 ± 0.90 ^b^	34.70 ± 1.02 ^a^	31.70 ± 0.88 ^c^	31.90 ± 0.86 ^c^	0.004
MUFA	44.10 ± 1.20 ^a^	43.00 ± 1.15 ^ab^	41.30 ± 1.18 ^b^	43.94 ± 1.10 ^a^	44.20 ± 1.12 ^a^	0.026
PUFA	24.50 ± 0.88 ^a^	24.40 ± 0.82 ^a^	24.00 ± 0.80 ^ab^	24.36 ± 0.90 ^a^	23.90 ± 0.84 ^b^	0.042
n-6 PUFA	22.78 ± 0.82 ^a^	22.76 ± 0.79 ^a^	22.45 ± 0.76 ^a^	22.46 ± 0.81 ^a^	22.18 ± 0.80 ^a^	0.81
n-3 PUFA	1.72 ± 0.11 ^b^	1.64 ± 0.10 ^b^	1.55 ± 0.09 ^b^	1.90 ± 0.12 ^a^	1.72 ± 0.11 ^b^	0.009
**Nutritional lipid indices**						
PUFA/SFA ratio	0.78 ± 0.04 ^a^	0.75 ± 0.03 ^ab^	0.69 ± 0.03 ^b^	0.77 ± 0.04 ^a^	0.75 ± 0.03 ^ab^	0.006
n-6/n-3 ratio	13.25 ± 0.72 ^bc^	13.87 ± 0.80 ^b^	14.49 ± 0.84 ^a^	11.81 ± 0.65 ^c^	12.87 ± 0.70 ^bc^	0.002
Atherogenicity index	0.37 ± 0.03 ^c^	0.41 ± 0.03 ^b^	0.46 ± 0.04 ^a^	0.40 ± 0.03 ^bc^	0.38 ± 0.03 ^c^	0.005
Thrombogenicity index	0.81 ± 0.05 ^bc^	0.84 ± 0.05 ^b^	0.92 ± 0.06 ^a^	0.78 ± 0.05 ^c^	0.80 ± 0.04 ^c^	0.017

Values are presented as mean ± standard deviation (SD). For fatty-acid measurements, the two birds sampled from each pen were considered subsamples, with the pen retained as the experimental unit (*n* = 4 pens per treatment). Within each row, means bearing different superscript letters differ significantly among dietary treatments (*p* < 0.05), whereas means sharing at least one superscript letter are not significantly different. Superscript letters are not shown when the overall treatment effect is not significant. T1 = control diet without BSFL meal; T2 = diet containing 5% standard full-fat BSFL meal; T3 = diet containing 10% standard full-fat BSFL meal; T4 = diet containing 10% polyphenol-enriched full-fat BSFL meal produced from larvae reared on olive leaves and spent tea residues; T5 = diet containing 10% defatted standard BSFL meal. BSFL = black soldier fly larvae; FA = fatty acids; SFA = saturated fatty acids; MUFA = monounsaturated fatty acids; PUFA = polyunsaturated fatty acids. The atherogenicity and thrombogenicity indices were calculated from the fatty-acid composition.

## Data Availability

The data used to support the findings of this study are included within the article. Any other data are available upon request.
